# Longitudinal clinical proteomics reveals pneumonia type–specific protein biomarkers and autoantibodies

**DOI:** 10.1172/jci.insight.203056

**Published:** 2026-06-08

**Authors:** Anna Semenova, Taylor A. Poor, Johannes B. Müller-Reif, Sai Rama Sridatta Prakki, Phillip Geyer, Martin Mück-Häusl, Rogan A. Grant, Luke Rasmussen, Lesca M. Holdt, Daniel Teupser, Matthias Mann, Ali Ö. Yildirim, Richard G. Wunderink, Alexander V. Misharin, Ben D. Singer, G.R. Scott Budinger, Theodore S. Kapellos, Herbert B. Schiller

**Affiliations:** 1 Research Unit for Precision Regenerative Medicine, Helmholtz Munich, Comprehensive Pneumology Center (CPC), Member of the German Center for Lung Research (DZL), Munich, Germany.; 2 Division of Pulmonary and Critical Care Medicine, Department of Medicine, Feinberg School of Medicine, Northwestern University, Chicago, Illinois, USA.; 3 Max Planck Institute of Biochemistry, Department of Proteomics and Signal Transduction, Martinsried, Germany.; 4 Institute of Computational Biology, Helmholtz Zentrum München, Munich, Germany.; 5 OmicEra Diagnostics GmbH, Planegg, Germany.; 6 Division of Health and Biomedical Informatics, Department of Preventive Medicine, Feinberg School of Medicine, Northwestern University, Chicago, Illinois, USA.; 7 Institute of Laboratory Medicine, University Hospital, LMU Munich, Munich, Germany.; 8 Institute of Lung Health and Immunity, Helmholtz Munich, CPC, Member of the DZL, Munich, Germany.; 9 Institute of Experimental Pneumology, LMU University Hospital, Ludwig-Maximilians University, Munich, Germany.

**Keywords:** Autoimmunity, Infectious disease, Inflammation, Autoimmune diseases, COVID-19, Influenza

## Abstract

Community-acquired pneumonia is a major cause of morbidity and mortality globally. Specific molecular endotypes are currently not well defined, and different viral or bacterial pathogens may trigger specific host responses and pathogenic mechanisms. We performed longitudinal proteomic profiling of bronchoalveolar lavage fluid and plasma from bacterial, influenza, and SARS-CoV-2–driven pneumonia. Our analysis revealed highly pneumonia type–specific proteomic signatures, including COVID-19–specific antibodies locally produced in the lung. These antibodies showed biased immunoglobulin V–domain usage, linked to a *CD69*/*CD83* plasma cell state associated with disease severity and degree of autoimmunity. Using mass spectrometry–driven autoantibody profiling in 2 independent COVID-19 cohorts, we identified 177 putative autoantibodies targeting extracellular matrix, nuclear, and immune-related proteins. Of note, temporal changes in autoantibody profiles correlated with clinical markers of inflammation, organ dysfunction, and duration of hospitalization. These findings highlight the autoimmune aspects of COVID-19 and provide potential biomarkers and therapeutic targets to help improve patient outcomes.

## Abstract 2

COVID-19 patients produce unique lung autoantibodies that track lung damage, distinguishing it from bacterial and viral pneumonias, and reveal autoimmune mechanisms driving severe disease.

## Introduction

Respiratory infections represent a major global health burden and the leading cause of death from infectious disease worldwide (1). Bacterial, viral, or fungal infections of the distal airways and alveoli directly damage the lung and induce local and systemic immune responses that present as the clinical syndrome of pneumonia. In severe pneumonia, damage to the alveolar-capillary barrier results in alveolar flooding and the acute respiratory distress syndrome, sometimes complicated by respiratory failure and death (2). Severe pneumonia and its complications are more common in the very young, the elderly, and immunocompromised (3).

The COVID-19 pandemic illustrated the devastating effect of infectious respiratory diseases on healthcare systems and national economies (4, 5). The large number of patients requiring hospitalization for severe COVID-19 provided an opportunity to understand pneumonia pathobiology and sequelae from the perspective of an individual pathogen and to compare it with pneumonia caused by other pathogens.

SARS-CoV-2 enters the lung through droplet aspiration, where it infects alveolar type 2 cells and alveolar macrophages, and is sustained by inflammatory signaling loops between activated T cells and alveolar macrophages that persists until the virus is cleared (6, 7). Antigen-specific T cell responses targeting key viral proteins are critical for viral clearance, but ineffective T cell responses drive immunopathology (8).

B cell–mediated responses also play an important role in COVID-19 pathobiology. Although antibodies against SARS-CoV-2 play a protective role by blocking viral entry via ACE2 receptor interaction (9), dysregulated humoral responses, characterized by extrafollicular B cell activation, low somatic hypermutation (10), Fc-mediated proinflammatory cytokine production, and auto-reactive antibodies, exacerbate immunopathology (11, 12).

Immune dysregulation and autoimmunity may contribute to acute COVID-19 but also play a role in the prolonged sequelae of COVID-19 reported in a substantial number of patients. For example, investigators have reported autoantibodies targeting type I IFNs (12), ACE2, G protein-coupled receptors, and components of the vascular and coagulation systems (13–16) in patients with COVID-19. Autoantibodies have also been reported against nuclear, phospholipid, cytoplasmic, immune signaling molecules, cardiac antigens, and extracellular matrix proteins; however, their clinical relevance remains incompletely understood. Transfer of immunoglobulins leads to postacute sequelae of COVID-19–like (PASC-like) symptoms in mice (17, 18), while autoantibodies against cardiolipin, platelet glycoprotein, and platelet factor 4 have been linked to thrombotic complications in acute disease (15, 19). Intriguingly, autoantibodies against chemokines correlate with protection from PASC (11). Many of them also occur in healthy individuals (20–23), highlighting a critical gap in our understanding of their pathogenic relevance.

We hereby employed an integrated multi-omics approach to dissect local and systemic immune responses in bronchoalveolar lavage fluid (BALF) and blood samples from patients with COVID-19 compared with patients with pneumonia secondary to other viruses or bacteria. Mass spectrometry detected pneumonia type–specific protein signatures in 2 independent patient cohorts, several also detectable in plasma. Single-cell RNA-seq (scRNA-seq) further identified COVID-19–specific immunoglobulin V–segment usage in peripheral blood linked to a distinct plasma cell state, enriched for autoimmune-related genes. Using a differential antigen capture (DAC) assay, we profiled circulating putative autoantibodies, several of which correlated with disease severity and hospitalization length. Collectively, our findings provide a comprehensive framework of pneumonia-associated autoantibodies and protein biomarkers that highlight their potential roles in COVID-19 pathophysiology.

## Results

### Mass spectrometry reveals pneumonia type–specific protein signatures in bronchoalveolar fluid.

**Figure 1:**
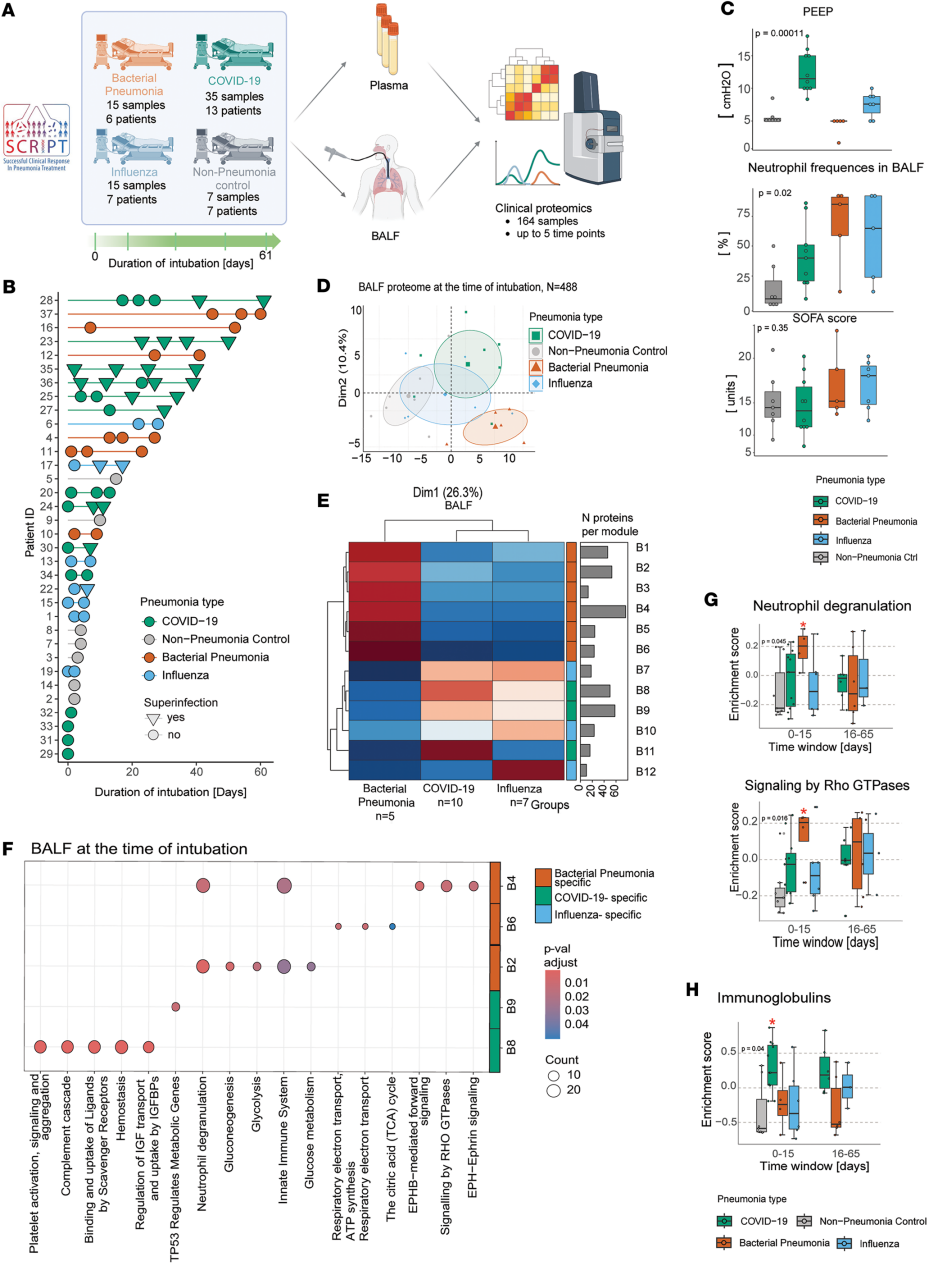
Pneumonia-specific signatures can be detected in BALF upon intubation in the intensive care unit. (**A**) Overview of study design. (**B**) Lollipop chart displaying patient numbers and the longitudinal resolution of sample collection per pneumonia type. (**C**) Box plots that compare clinical parameters and phenotypical observations across pneumonia types. Data are represented as mean ± SD, and variance was statistically assessed with the non-parametric Kruskal-Wallis test. (**D**) Principal component analysis of BALF proteomes at the time of intubation based on the 488 most variable proteins. The 20% most variable proteins were calculated by ranking the BALF proteome by decreasing variance across all pneumonia type specimens. (**E**) Hierarchical clustering of 12 BALF pneumonia-specific protein modules at day 0 of intubation as derived from coexpression network analysis. The color scale denotes the mean fold change of the proteins in each module. (**F**) Pathway analysis of selected BALF module proteins at intubation induction. The top 5 enriched Reactome terms for each module are displayed. Color code depicts the adjusted *P* value, and point size refers to the number of proteins detected per term. (**G** and **H**) Box plots displaying the longitudinal enrichment of neutrophil degranulation (**G**) and immunoglobulin (**H**) Reactome terms in BALF specimens across pneumonia types. Each dot represents the enrichment score for an individual patient. Data are represented as mean ± SD, and the variability between groups was statistically assessed with the nonparametric Kruskal-Wallis test. Proteins involved in the term are displayed on the right side of the plot.

To identify pneumonia type–specific biomarkers, we sampled BALF and plasma from individuals enrolled in the Successful Clinical Response in Pneumonia Therapy (SCRIPT) study at Northwestern Memorial Hospital in Chicago (hereafter referred to as the “Chicago cohort”). The cohort encompassed control donors (*n* = 7) and patients requiring mechanical ventilation in the intensive care unit (ICU) who were diagnosed with pneumonia of bacterial (*n* = 6), influenza (*n* = 7), or COVID-19 (*n* = 13) origin (Supplemental Table 1; supplemental material available online with this article; https://doi.org/10.1172/jci.insight.203056DS1). Sample collection occurred at the earliest within 48 hours of intubation, and collection continued over the entire length of ICU stay, reaching a maximum of 5 time points per patient over up to 60 days after intubation (Figure 1, A and B). Pneumonias of different origins were substantially different in their clinical presentation and host immune responses, as exemplified by the significantly higher (*P* = 0.00011) positive end-expiratory pressure (PEEP) in COVID-19 and neutrophil relative frequencies (*P* = 0.02) in bacterial pneumonia. In contrast, the sequential organ failure assessment (SOFA) score was similar across groups, suggesting a comparable degree of tissue damage across pneumonia types (Figure 1C).

We next sought to understand how the clinical groups are different on the molecular level. We applied mass spectrometry–based proteomics to BALF specimens from 32 patients, which quantified 985 ± 275 proteins. Principal component analysis (PCA) of the first time-point measurements showed that the proteome profiles of different pneumonia types are well separated already at baseline (Figure 1D and Supplemental Figure 1, A and B); bacterial pneumonia specimens were distinguished by PC1, whereas COVID-19 pneumonias were separated by PC2. The 488 most variable proteins were then used to construct a coexpression network analysis of the BALF proteomes to identify features that drive the heterogeneity in pneumonia types.

Proteins with similar expression patterns were grouped into modules and were visualized in a heatmap (Figure 1E). Hierarchical clustering identified 12 protein modules (6 bacterial pneumonia–specific, 3 COVID-19–specific, 3 influenza-specific), which we further interrogated using pathway analysis. Proteins belonging to the bacterial pneumonia–specific modules were enriched, among other biological processes, in neutrophil degranulation (module B2), signaling by Rho GTPases (module B4), and respiratory electron transport (module B3) (Figure 1F, Supplemental Figure 1C, and Supplemental Table 2). Consistent with these findings, the abundance of neutrophil degranulation proteins correlated positively (*r* = 0.74; *P* = 0.001) with BALF neutrophil frequencies in bacterial pneumonia (Supplemental Figure 1D). COVID-19–specific modules were enriched in the regulation of the complement cascade (module B8), platelet degranulation (module B8), p53 metabolic regulation (module B9), and immunoglobulins (modules B8, B9, B11) (Figure 1F, Supplemental Figure 1E, and Supplemental Table 3).

To determine how these proteomic signatures were regulated longitudinally, we also analyzed their enrichment at later time points (Figure 1, G and H, and Supplemental Figure 1, F and G). During the early interval (days 1–15), bacterial pneumonia was uniquely characterized by significant enrichment of neutrophil degranulation and Rho GTPase effector pathways, which lost statistical significance at later time points (Figure 1G). In contrast, the COVID-19 pneumonia cases showed a pronounced increase in immunoglobulin-related, complement, and platelet activation signatures that remained persistently elevated also at later time points compared with the other groups (Figure 1H and Supplemental Figure 1, F and G).

Taken together, we identified BALF protein signatures that distinguish between bacterial, viral, and COVID-19 pneumonia types. Patients with COVID-19 exhibited persistently increased levels of complement cascade proteins, platelet aggregation markers, and a specific set of immunoglobulins.

### A COVID-19–specific set of antibodies locally enriched in the lung versus blood.

The BALF proteome can be derived either by local production in the lung or by leakage of plasma factors into the lung due to acute injury and barrier defects. We made use of the fact that we collected matched BALF and plasma samples to analyze the relative enrichment of proteins in the lung versus the blood.

**Figure 2:**
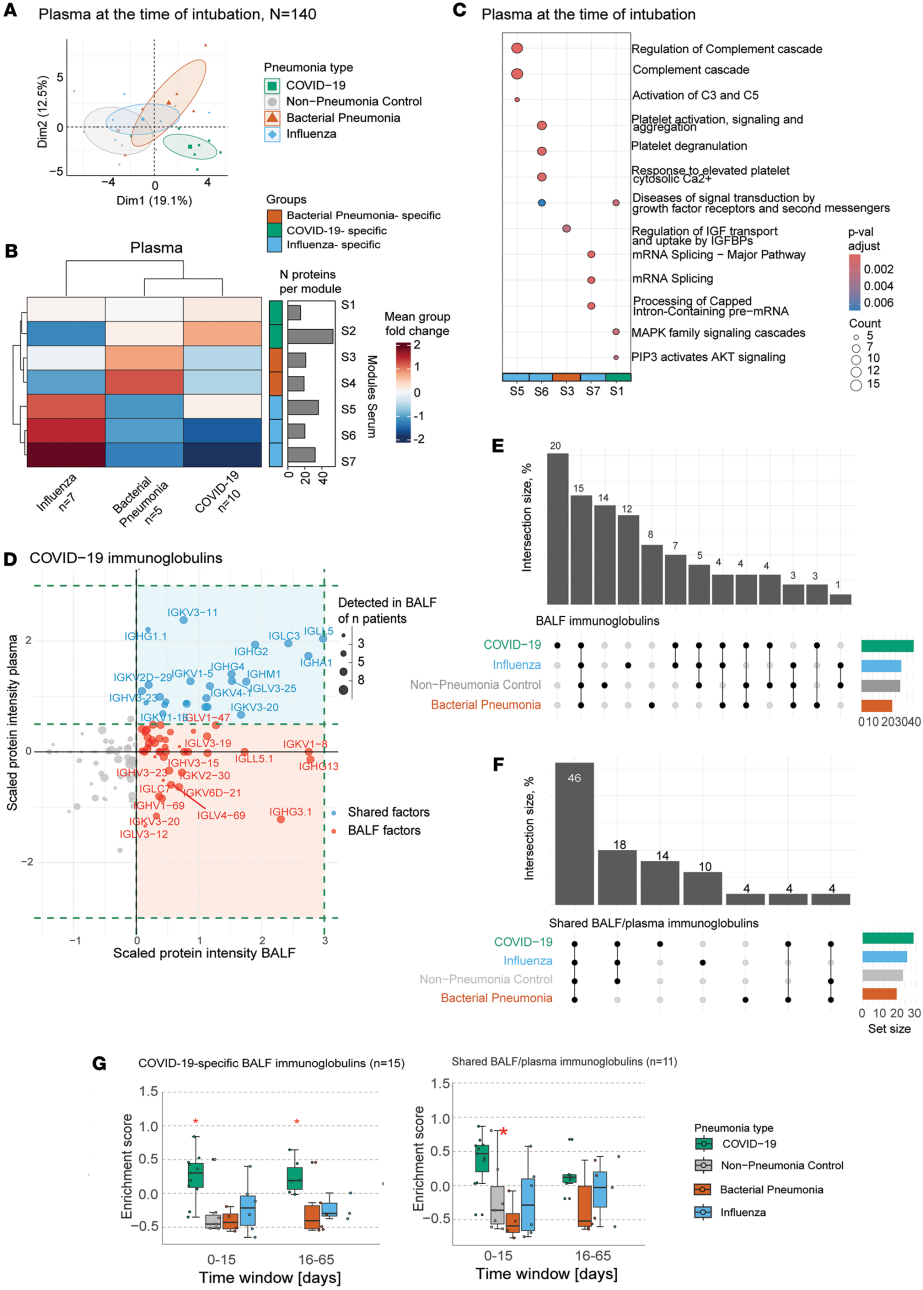
Integrative analysis of body fluid proteomes reveals lung-resident biomarkers. (**A**) Principal component analysis of plasma proteomes at the time of intubation induction based on the 140 most variable proteins. We calculated the 20% most variable proteins by ranking the plasma proteome by decreasing variance across all pneumonia type specimens. (**B**) Hierarchical clustering of 7 plasma pneumonia-specific protein modules at day 0 of intubation as derived from coexpression network analysis. The color scale denotes the mean fold change of the proteins in each module. (**C**) Pathway analysis of selected plasma module proteins at intubation induction. The top 5 enriched Reactome terms for each module are displayed. Color code depicts the adjusted *P* value, and point size refers to the number of proteins detected per term. (**D**) Scatter plot displaying immunoglobulin segment abundance in the BALF (*x* axis) and plasma (*y* axis) of patients with COVID-19 at the time point upon intubation. Dot size corresponds to the number of patients that express a detected protein. (**E** and **F**) Upset plot displaying the overlap of BALF and shared BALF/plasma immunoglobulin segments across pneumonia types. (**G**) Box plots displaying the longitudinal enrichment of COVID-19–specific BALF and shared BALF/plasma immunoglobulin signatures across pneumonia types. Each dot represents the enrichment score for an individual patient. Data are represented as mean ± SD and were statistically assessed with the nonparametric Kruskal-Wallis test. *P* < 0.05.

Using mass spectrometry, we quantified 374 ± 33 proteins in the plasma samples. The PCA of the baseline time point measurements revealed a clear separation of the COVID-19 proteomes by PC1 and PC2 (Figure 2A and Supplemental Figure 2, A and B). Similarly to BALF, coexpression network analysis of the plasma proteomes grouped the proteins in 7 modules (2 bacterial pneumonia–specific, 2 COVID-19–specific, 3 influenza-specific) (Figure 2B and Supplemental Table 4). Proteins belonging to the bacterial pneumonia–specific modules were enriched in the regulation of insulin-like growth factor transport and uptake (module S3) (Figure 2C). COVID-19–specific modules included immunoglobulins (module S2). Lastly, influenza-specific modules displayed strong immune responses related to complement cascades (module S5), platelet activation (module S6), and mRNA splicing (module S7) (Figure 2C, Supplemental Figure 2C, and Supplemental Table 5).

In both the BALF and plasma, we found COVID-19–specific enrichment of immunoglobulins. Since we measured both biofluids from matched donors, we were able to quantitatively compare the enrichment of proteins that were detected in both the BALF and the plasma (Figure 2D). Indeed, in the quadrant that represents proteins with high abundance in both compartments, we found a set of antibodies (V-segments) that were highly abundant in both BALF and plasma (highlighted in blue) (Figure 2D). In addition, several antibodies were quantitatively enriched in BALF and, hence, likely produced locally (highlighted in red). Interestingly, many locally produced V-segments were highly specific to COVID-19 (20%), influenza (12%), or bacterial pneumonia (8%) (Figure 2E), in contrast to the systemic V-segments, which were less specific to individual pneumonia — COVID-19 (14%), influenza (10%), bacterial pneumonia (4%) — types (Figure 2F and Supplemental Figure 2D). Longitudinal assessment of immunoglobulin profiles across pneumonia types confirmed the higher enrichment of COVID-19–specific BALF and shared BALF/plasma segments, evident at both time intervals and significantly pronounced at days 0–15 (Figure 2G).

In summary, our plasma proteomics analysis identified distinct molecular signatures across pneumonia types. In COVID-19, immunoglobulin segments were enriched in both BALF and circulation, exhibiting specificity and longitudinal persistence.

### Clonal expansion of a specific set of plasma cells in severe COVID-19.

**Figure 3:**
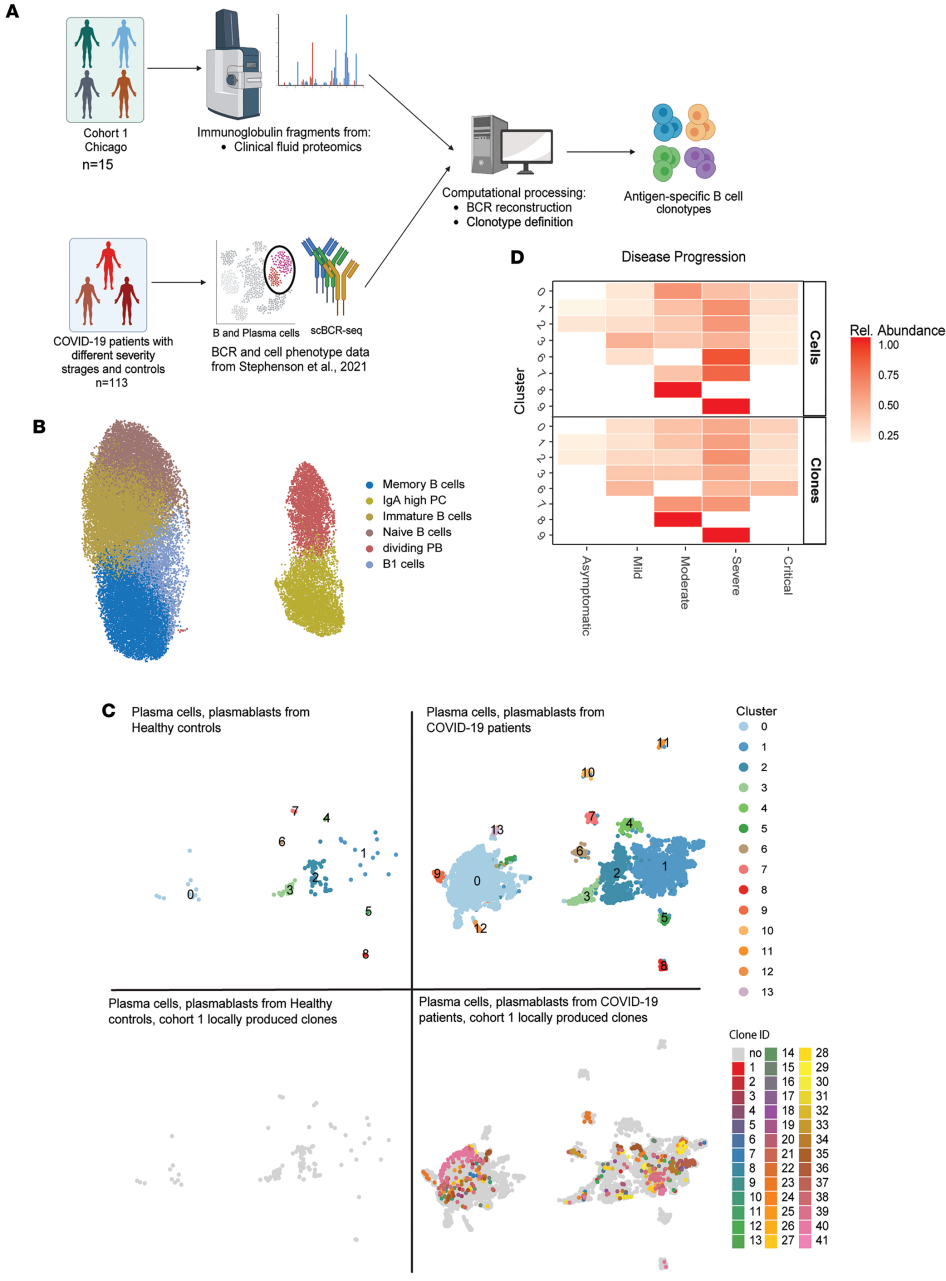
Peripheral B cell clonal dynamics are associated with COVID-19 severity. (**A**) Overview of bioinformatics workflow. (**B**) UMAP visualization of 38,063 peripheral blood B/plasma cells from 90 patients with COVID-19 and 23 healthy controls, colored by cluster identity. (**C**) UMAP projection of 8,599 plasma cells from 90 patients with COVID-19 and 23 healthy controls. Lower panels show clonal expansions of BALF-derived V-fragment lineages mapped to patients with COVID-19 vs controls. (**D**) Heatmap visualizing the relative abundance of B/plasma cell molecular states across stages of COVID-19 disease progression.

We next verified the observed COVID-19–specific antibody V-segment bias in an independent multimodal scRNA-seq dataset of peripheral blood samples (24), encompassing healthy controls (*n* = 23), as well as patients representing asymptomatic (*n* = 9), mild (*n* = 23), moderate (*n* = 30), severe (*n* = 13), and critical (*n* = 15) stages of COVID-19 (Figure 3A) (24). We reclustered a total of 38,063 B cells and plasma cells (Supplemental Figure 3A), with B cells forming 6 distinct clusters upon consideration of both their transcriptomes (Supplemental Figure 3B) and surface proteomes (Supplemental Figure 3, C and D). Annotation of the resulting clusters using canonical marker genes (25) identified memory, immature, naive, and natural B1 cells, as well as dividing plasmablasts and IgA-high plasma cells (Figure 3B and Supplemental Table 6). Notably, we observed an increase in the IgG and IgA relative proportion in B/plasma cells from severe disease (Supplemental Figure 3E), which was reflected in the significant (*P* = 0.01) increase in the relative frequencies of IgA-high plasma cells (*P* = 6.6 × 10^–6^) and dividing plasmablasts across COVID-19 stages. In contrast, naive (*P* = 0.00011) and memory B cells (*P* = 6.2 × 10^–5^) displayed the opposite pattern (Supplemental Figure 3E).

**Figure 4:**
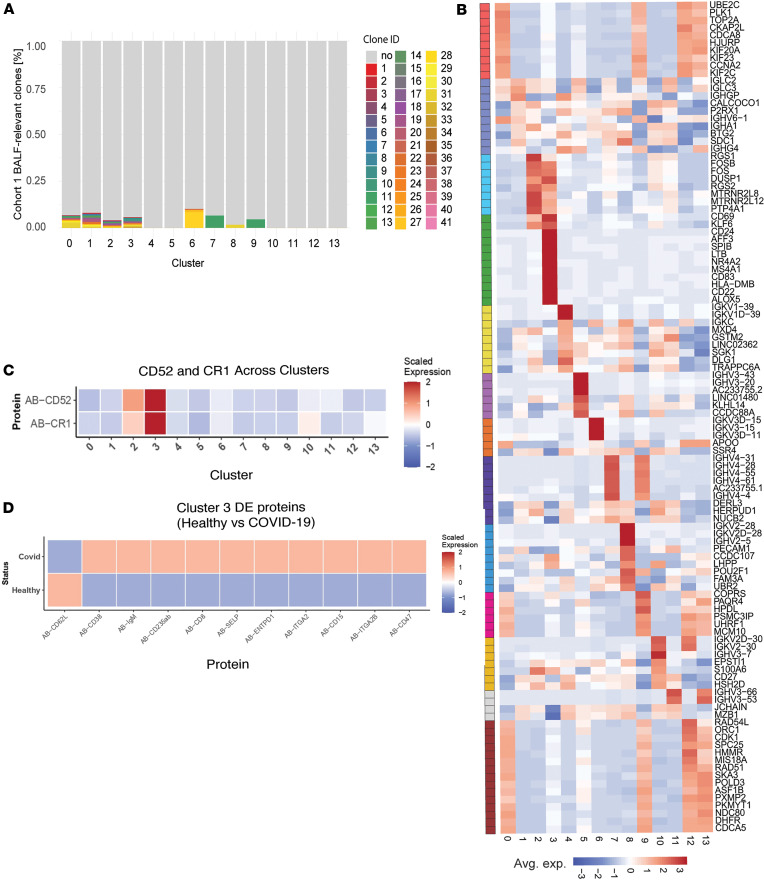
Distinct plasma cell states in patients with COVID-19 display autoimmune transcriptional signatures. (**A**) Bar plot showing clonal percentage of proteomics-identified V-segments per plasma cell state. (**B**) Heatmap of top differentially expressed genes for each plasma cell state. Color code denotes the scaled average expression across clusters. (**C**) Heatmap of top differentially expressed proteins for plasma cell state 3. Color code denotes the scaled average expression across clusters. (**D**) Heatmap of differentially expressed surface protein markers in plasma cell state 3 between healthy controls and COVID-19 donors.

BCR-seq analysis revealed that the COVID-19–specific V-segment clonotypes identified in our proteomics dataset were predominantly expressed by plasma cells (Supplemental Figure 3F). To explore this further, we reclustered these cells into 14 transcriptomically distinct molecular states (Figure 3C and Supplemental Table 7). These plasma cell states were the main source of BALF-derived V-fragments, highlighting their potential role in shaping COVID-19–specific humoral responses. The abundance and clonality expression of plasma states 0–3 progressively increased with severity with advancing disease severity, while others (cluster 6–9) were enriched in moderate and severe cases (Figure 3D). Notably, these plasma cell states were also the main source of the COVID-19–specific BALF immunoglobulins, highlighting their potential role in shaping disease-relevant humoral responses (Figure 4A).

Our findings prompted us to perform differential gene expression analysis to reveal how these states are different from each other (Figure 4B and Supplemental Table 7). As expected, light and heavy chain V-segments distinguished several plasma cell states. Plasma cell states 1 and 13 were characterized by the high expression of proliferation markers, whereas molecular state 3 represented activated plasma cells, defined by high expression of *CD69* and *CD83*. This plasma cell state has previously been marked by the expression of genes implicated in autoimmune diseases (26, 27). For instance, *NR4A2* and *LTB* drive the formation of ectopic lymphoid structures and pathological B cell responses in rheumatoid arthritis (27), while *AFF3* is a susceptibility factor for RA and type I diabetes (28). Additionally, the surface proteins such as *CD52* and *CR1*, which were upregulated in cluster 3, are also implicated in autoantibody production in B cell subsets from patients with rheumatoid arthritis and systemic lupus erythematosus (Figure 4C and Supplemental Table 8) (29, 30). Lastly, analysis of the plasma cell state 3 proteome showed an upregulation of markers, including CD38, ITGA2, ITGA2B, and SELP, suggesting enhanced interactions with the extracellular matrix and the vascular endothelium, as well as localized immune responses (Figure 4D and Supplemental Table 8).

Conclusively, we identified transcriptomically distinct plasma cell states that expand with COVID-19 severity and serve as the predominant source of the disease-specific antibody V-segments detected by proteomics. Notably, one of these states exhibited an activated, autoimmune-like transcriptional profile and featured genes implicated in ectopic lymphoid formation and autoantibody production.

### A differential autoantigen capture assay reveals COVID-19–specific autoantibodies.

**Figure 5:**
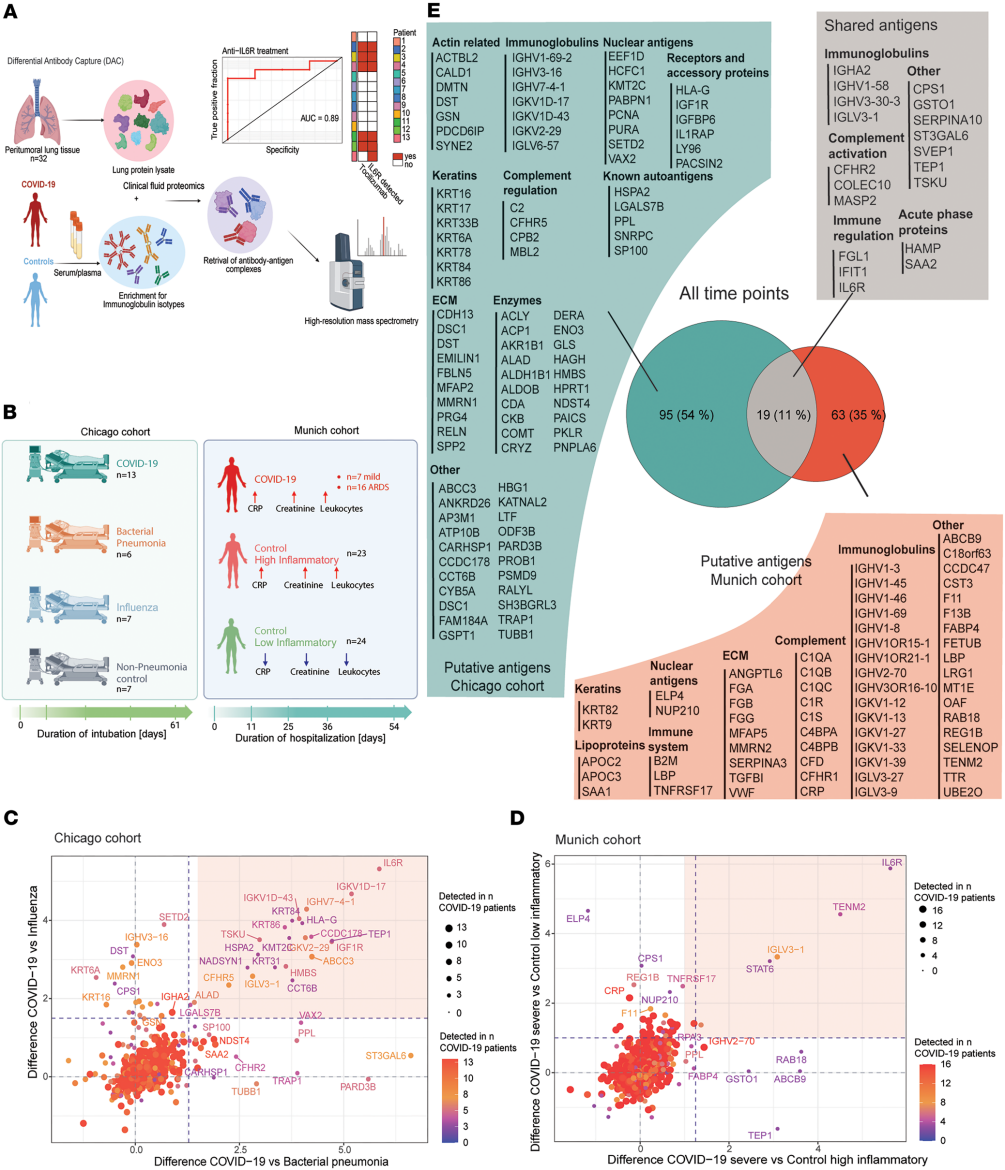
Comparative analysis of the putative autoantigen repertoire of 2 independent COVID-19 cohorts. (**A**) Flowchart of the DAC assay and benchmarking processes with anti–IL-6R antibody. (**B**) Schema of Chicago and Munich cohort demographics. (**C**) Scatter plot displaying fold changes of individual antigens in patients with COVID-19 (*n* = 13) of the Chicago cohort versus bacterial pneumonia patients (*n* = 6) on the *x* axis and influenza patients (*n* = 7) on the *y* axis at the first time point upon intubation. Dot size and color represent the number of patients with COVID-19 with a particular plasma autoantibody. Enriched putative autoantibodies in COVID-19 over bacterial pneumonia and influenza are depicted in the highlighted rectangle. (**D**) Scatter plot displaying fold changes of individual antigens in patients with severe COVID-19 (*n* = 16) of the Munich cohort versus high-inflammatory control patients (*n* = 23) on the *x* axis and low-inflammatory control patients (*n* = 24) on the *y* axis at the first point upon in hospitalization. Dot size and color represent the number of patients with COVID-19 with a particular plasma autoantibody. Enriched putative autoantibodies in COVID-19 over both controls are depicted in the highlighted rectangle. (**E**) Venn diagram demonstrating putative autoantibody repertoires of the Chicago and Munich cohorts grouped by molecular function. Displayed autoantigens are significantly enriched at least at 1 time point over at least 1 control group and in at least 3 patients with COVID-19.

Given the mounting evidence for autoantibodies in acute COVID-19, as well as PASC, and our observation that severe COVID-19 is associated with a specific V-segment bias, which could favor autoimmunity, we were interested in profiling autoreactive antibodies in our cohort. We recently developed a DAC assay (31), which is based on immunoprecipitation of natively extracted human lung proteins using patient antibodies captured from their plasma or serum (Figure 5A). Mass spectrometry quantifies the relative antibody-mediated enrichment of proteins in each patient compared with the average in a control population. Thus, we used the DAC assay to analyze antibody-mediated autoreactivities in the Chicago cohort alongside a second longitudinal cohort of patients with COVID-19 (hereafter referred to as the “Munich cohort”), which included mild cases (*n* = 7) and ICU-admitted patients with severe COVID-19 (*n* = 16) (Figure 5B and Supplemental Table 9). The Munich cohort also included 2 COVID-19 negative control groups, categorized as low inflammatory (*n* = 24) and high inflammatory (*n* = 23) based on the circulating levels of CRP, creatinine, and leukocytes (Supplemental Figure 4A). Importantly, BALF and shared BALF/plasma V-segments were significantly enriched over time in patients with severe COVID-19 compared with mild cases and the controls, substantiating the presence and persistence of pneumonia-specific immunoglobulins throughout disease progression (Supplemental Figure 4, B and C).

In our previous study, the DAC assay reached almost 100% sensitivity and specificity against a clinical ELISA benchmark (31). To test these performance metrics in the present study, we made use of the fact that some patients were treated with the anti–IL-6R therapeutic monoclonal antibody Tocilizumab (32). We observed that IL-6R was significantly enriched over controls only in patients who received the therapeutic antibody (AUC = 0.89) (Figure 5A), demonstrating the high specificity of the immunoprecipitation. To identify putative COVID-19–specific autoantigens in severe disease, patients in the Chicago cohort and severe cases from the Munich cohort were screened, and the enriched antigens in each cohort at the first time point were further analyzed (Figure 5, C and D, and Supplemental Figure 4, D and E). In both cohorts, we found antigens significantly enriched over other pneumonia types (Figure 5C and Supplemental Figure 4D) or controls (Figure 5D and Supplemental Figure 4E) already upon hospitalization, with a prevalence ranging from 3 to 8 patients across all putative autoantigens.

Overall, we identified 95 putative autoantibodies in the Chicago cohort and 63 putative autoantibodies in the Munich cohort, ranging with functions from extracellular matrix proteins, keratins, lipoproteins, complement cascade members, nuclear antigens, enzymes, receptors, acute phase proteins, and signal transduction proteins (Figure 5E and Supplemental Table 10). Several of these antibodies have been associated with autoimmune disease. For example, phospholipid (PPL) autoantibodies were described in pulmonary fibrosis donors (33), anti-U1 snRNP (SNRPC) have been linked to mixed connective tissue disease and SLE (34), whereas anti-SP100 are associated with primary biliary cholangitis (35). Despite the substantial interindividual variance and significant differences between the Chicago and Munich cohorts, we also identified 19 hits (15 putative autoantigens, 4 immunoglobulins) that were shared between cohorts and significantly enriched in at least at 1 time point over at least 1 control group and in at least 3 patients with COVID-19 with severe disease (Figure 5E). Among the shared hits, autoantibodies against acute phase response proteins, complement, and anti-glutathione S-transferase omega-1 (GSTO1) have been described in autoimmune diseases, SLE, RA, and COVID-19, leading to excessive organ damage (36–38). Notably, the discovery of IFIT1 autoantibodies in our data supports previous work that has described autoantibodies interfering with IFN signaling in patients with severe COVID-19 (12, 39).

In conclusion, we detected antibodies with an affinity for native lung proteins in the plasma of patients with COVID-19 from 2 independent cohorts, suggesting the presence of multiple putative autoantigens in severe COVID-19. Many of these shared and cohort-specific antibodies encompass extracellular matrix proteins, immunoglobulins, complement cascade members, acute phase proteins, and others, highlighting both the diversity and clinical relevance of COVID-19–associated autoimmunity and underscoring the value of unbiased proteomics in revealing biomarkers for predicting disease severity.

### COVID-19 patient clustering reveals temporal and severity-associated dynamics in autoantibody responses.

**Figure 6:**
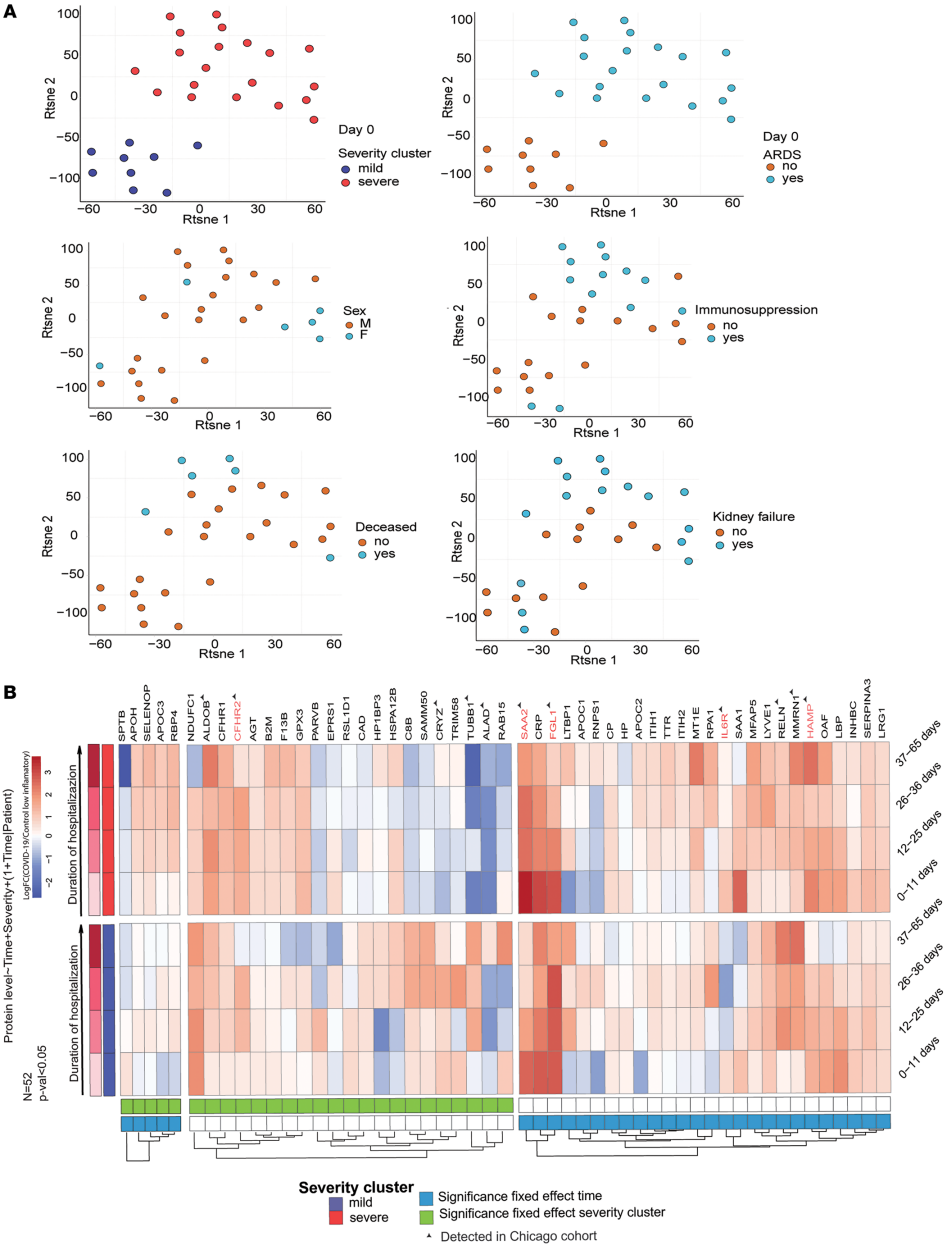
Longitudinal profiling of autoreactivities in mild and patients with severe COVID-19. (**A**) t-SNE of 27 patients with COVID-19 enrolled in the Ludwig-Maximilians-University Hospital (Munich cohort) based on available clinical parameters at the beginning of intubation. Two clusters (mild and severe) are shown. (**B**) Heatmap of mixed-effects model for time and COVID-19 severity. Only significant proteins (*P* < 0.05) are displayed, and shared proteins with the Chicago cohort are highlighted. Color code represents average expression in time intervals after hospitalization.

Since the Chicago cohort encompassed severe COVID-19 samples, we set out to compare the longitudinal profiles in both the severe and mild cases of the Munich cohort. We first performed patient clustering based on all available clinical parameters. This revealed 2 patient groups; cluster 1 comprised individuals with severe disease manifestations, including acute respiratory distress syndrome (ARDS), immunosuppression, a higher incidence of kidney failure, and increased mortality, whereas cluster 2 included patients with milder symptoms (Figure 6A). These clinical differences were mirrored in the plasma levels of IL-6 and CRP, biomarkers of systemic inflammation (Supplemental Figure 5, A and B). To characterize temporal variations in autoantibody abundance, we subsequently grouped the observations into 3 time windows (days 0–11, 12–36, and 37–56) (Supplemental Figure 5C) and applied a mixed-effects linear model, with time and severity as fixed effects and patient ID as a random effect. The generated model identified 52 autoantigens with significant associations with 1 or both fixed effects (Figure 6B).

Among the identified autoantigens, 5 were significantly associated with both time and severity. In particular, antibodies against APOH, SELENOP, APOC3, and RBP4 were prevalent in patients with severe COVID-19, and their levels increased during hospitalization. Within the putative autoantibodies that were associated with disease severity, severe patients had elevated levels of anti-F13b, anti-B2M, anti-AGT, and anti-GPX3 antibodies compared with mild cases, whereas mild patients had higher levels of anti-TUBB1, anti-ALAD, anti-RAB15, anti-TRIM58, anti-CRYZ, anti-CD8B, anti-SAMM50, anti-PARVB, anti-CAD, ant-RSL1D1, and anti-EPRS1 antibodies (Figure 6B). Lastly, we detected putative autoantigens that were associated with hospitalization duration in both mild and severe patients. Notably, antigens that were correlated in both severity groups (SAA1, SAA2, LBP, FGL1) were related to acute inflammation and decreased over time. In contrast, those correlating in severe cases (IL-6R, CRP) showed both upward and downward trends over time (Figure 6B).

Taken together, we followed the dynamics of putative autoantibodies in the circulation of a longitudinal cohort of patients with COVID-19, and we described immunoglobulins that display temporal or severity-related abundance patterns.

### Autoreactivities correlate with clinical parameters and severity in patients with COVID-19.

**Figure 7:**
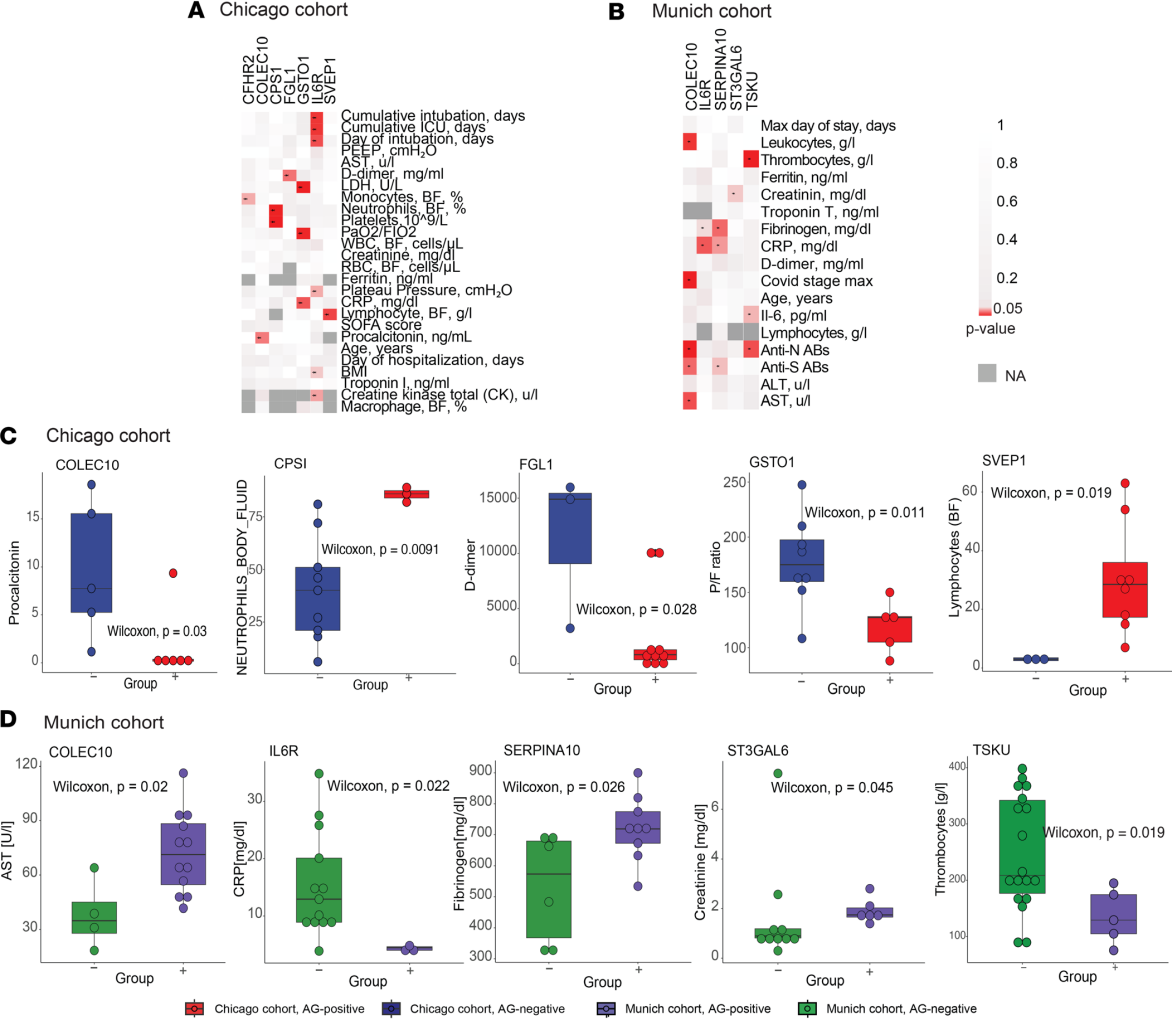
Blood putative autoantibodies are associated with clinical parameters in 2 independent severe COVID-19 cohorts. (**A** and **B**) Heatmaps presenting associations between the detection antibodies against shared putative antigens (*n* = 15) in the peripheral blood of (**A**) *n* = 13 patients with COVID-19 of the Chicago and (**B**) *n* = 16 patients with severe COVID-19 of the Munich cohort upon intubation on the *x* axis and selected clinical parameters on the *y* axis in the Chicago and Munich cohorts. The color denotes the output of the Wilcoxon test (*P* value). Dark gray color indicates detection in less than 3 patients or missing clinical data, preventing statistical comparison. (**C** and **D**) Box plots showing the significant associations between 19 putative shared autoantigens and clinical parameters in 2 cohorts: Chicago (**C**) and Munich (**D**). The *x* axis represents patients with COVID-19 categorized based on the presence (+) or absence (–) of detected autoantigens at the time of intubation. Statistical significance was assessed using the Wilcoxon test.

We subsequently assessed the abundance of the identified autoreactivities related to key clinical features that included measures of lung function (PaO_2_/ FiO_2_ ratio [P/F ratio], plateau pressure, PEEP, SOFA score), inflammation, and antiviral immunity (CRP, ferritin, procalcitonin, IL-6, anti-N antibodies, anti-S antibodies), coagulation and thrombosis (D-dimer, fibrinogen, platelet counts), multiorgan injury (ALT, AST, LDH, troponin I, troponin T, creatinine, creatine kinase), disease severity and prognosis (maximum COVID stage, cumulative ICU days, intubation days, BMI, age), and leukocyte composition (neutrophil, macrophage, lymphocyte counts). In both cohorts, we identified a broad spectrum of associations between the putative autoantibodies and clinical parameters upon admission to the hospital. The assessment of associations between the shared autoantibodies in the Chicago and Munich cohorts and clinical parameters revealed a few putative associations with the number of days in intubation, coagulation, liver enzymes, antiviral inflammation, as well as blood leukocyte and platelet abundance (Figure 7, A and B).

In both cohorts, we observed a broad range of associations between the putative autoantigens and clinical parameters at the time of hospital admission (Figure 7, A and B). Some of the most prominent shared patterns linked autoreactivities to prolonged intubation, coagulation abnormalities, elevated liver enzymes, antiviral responses, and altered blood cell counts. Within the shared autoantigens, several targets (IL-6R, TSKU, CPS1, FGL1, SVEP1, CFHR2, ST3GAL6, GSTO1, SERPINA10, COLEC10) demonstrated correlations, spanning respiratory compromise (PaO_2_/FiO_2_ ratio, plateau pressure, D-dimers), systemic inflammation (IL-6, CRP, procalcitonin), coagulation activation (fibrinogen, platelet counts), tissue injury (LDH, AST, creatinine, creatine kinase), clinical severity (maximum COVID-19 stage, BMI, intubation duration), antibody responses (anti-N, anti-S), and immune cell counts (leukocytes, monocytes, neutrophils, lymphocytes).

In addition, we detected cohort-specific patterns. In the Chicago cohort, autoreactivities were directed, among others, at lung structural proteins (KRT6A, KRT78, KRT84, KRT86), desmosomes (DST, PPL, DSC1), the actin cap (GSN, CALD1, ACTBL2, HCFC1), and meiotic synapses (HSPA2, SYNE2). These autoantibody profiles were associated with patient outcomes, encompassing respiratory function (P/F ratio, PEEP, plateau pressure), markers of inflammation and tissue injury (procalcitonin, LDH, creatine kinase, D-dimers), cell frequencies (lymphocyte, platelet, and total blood cell counts), and disease burden (intubation, duration of hospitalization, ICU stay, BMI, age) (Supplemental Figure 6A). In the Munich cohort, distinct autoreactivities targeting proteins, including ABCB9, RAB18, REG1B, NUP210, and ANGPTL6 were associated with changes in thrombocyte counts, ferritin and creatinine levels, the maximum COVID-19 stage, D-dimer and IL-6 levels, and antiviral antibody titers (Supplemental Figure 6B).

To test whether the presence of shared autoantibodies was associated with differences in clinical status, we compared the values of the aforementioned clinical parameters — including indicators of lung function, systemic inflammation, coagulation activity, and multiorgan injury — between patients positive and negative for the shared autoreactivities across both cohorts, and we performed the same for the cohort-specific autoreactivities (Figure 7, C and D, and Supplemental Figure 6, C and D). This analysis showed that antibody-positive patients often displayed more severe impairments in, and higher indices of, disease severity than antibody-negative patients, reinforcing the link between specific autoantibody profiles and the clinical manifestations of severe COVID-19.

To summarize, our analysis revealed clear associations between the levels of circulating autoantibodies and multiple clinical parameters in patients with COVID-19. Based on the biological functions of their target antigens, the detected autoantibodies may disrupt key molecular pathways, contributing to multiorgan injury and coagulopathy, commonly observed in severe cases. Moreover, their presence may prolong intubation and hospitalization requirements and increase susceptibility to secondary bacterial infections.

## Discussion

The specificity of cellular and molecular processes and the associated biomarkers in COVID-19 compared with the sequelae of other severe respiratory infections is not yet well established. Our study builds a solid framework of COVID-19–specific biomarkers in comparison with influenza and bacterial-mediated pneumonia using longitudinal proteomic profiling of both BALF and blood. In particular, we identified a COVID-19–specific set of plasma cell clonotypes and their corresponding antibodies that may contribute to the observed increase in B cell–mediated autoimmunity in severe COVID-19 and possibly its long-term sequelae (long-COVID). We found that autoantibody abundance correlated with both hospitalization duration and disease severity, and it was further associated with critical clinical indicators, including lung function decline, disease progression, leukocyte counts, coagulation abnormalities, systemic inflammation, and multiorgan failure. Whether these autoantibodies are pathogenic or biomarkers of disease severity will require prospective validation.

As expected, we observed distinct differences in clinical presentation between pneumonia types. These clinical patterns were mirrored in pneumonia type–specific biological pathways (40, 41). Importantly, these proteomic patterns persisted throughout the patient intubation period, underscoring the distinct pathophysiological mechanisms governing each pneumonia type. Importantly, key biological processes were consistently detected in both BALF and plasma, suggesting that loss of endothelial barrier integrity allows lung-derived proteins to leak into the circulation, thereby promoting inflammation and obstructing recovery (42–45). This highlights the potential of these blood proteomic signatures as clinically relevant biomarkers.

Humoral responses, an essential component of the adaptive immune system, are responsible for recognizing and neutralizing external threats. In healthy individuals, both naive and memory B cell compartments may harbor autoreactive clones under tight regulatory control. However, in severe inflammatory conditions, such as in COVID-19, SLE, and other autoimmune disorders, autoreactive, age-associated, and atypical B cell populations can expand and secrete pathogenic autoantibodies (46–50). Following SARS-CoV-2 infection, B cells initiate IgM production, followed by IgG and IgA responses that can persist for over a year (51, 52). However, in severe cases, germinal center formation is impaired, promoting extrafollicular B cell responses that favor rapid differentiation into plasma cells (10, 53) and the production of low-affinity, broad autoreactive antibodies. These antibodies often target nuclear antigens, phospholipids, and carbamylated proteins (16, 25, 54) and are linked to poor clinical outcomes (16). While autoreactive plasma cells typically contract during convalescence, serological autoreactivity can persist in some patients and may contribute to PASC (55, 56). In particular, antibodies with altered glycosylation or fucosylation patterns can bind to Fcγ receptors on myeloid cells, triggering excessive proinflammatory cytokine release (57). Lastly, cross-reactive B cell clonotypes that recognize both viral and self-antigens may play a dual role in antiviral defense and autoimmunity (58).

We found that the abundance of immunoglobulin fragments in COVID-19 BALF was increased across severity stages, and we identified a transcriptionally distinct population of activated plasma cells that expressed a broad repertoire of these proteomics-matched clonotypes. These cells expressed *CD69* and *CD83* and showed transcriptomic signatures linked to autoimmune diseases such as rheumatoid arthritis, type I diabetes, and SLE. CD69 engagement has been linked to TGF-β production, whereas its deficiency exacerbated inflammation in a murine collagen-induced model of arthritis (59), suggesting a dual role in regulating inflammation. This population may therefore constitute a putative target to mitigate autoimmunity progression without compromising antiviral immunity (47), while whether it could serve as a predictive biomarker for postinfectious autoimmunity in PASC remains to be determined and should be the focus of future longitudinal studies.

The discovery of plasma cell molecular states associated with autoimmunity prompted us to apply our mass spectrometry–based DAC assay, which allows unbiased and multiplexed identification of antibody-mediated autoreactivities (31). Using a mixed-effects model to disentangle the effects of disease severity and intubation time, we identified autoantibodies that target components of the type I IFN pathway (12), as well as antiphospholipid antibodies, also implicated in aberrant coagulation in a mouse model (60). Additionally, we found autoantibodies against cytokines and other immune signaling molecules (39), although we did not detect anti-chemokine autoantibodies, likely due to the limited cohort size and patient-specific heterogeneity in the autoantibody repertoire (11). Extending previous findings (60–62), we detected autoantibodies directed against C1q, B2GP1, SERPINA10, and SVEP1, which are linked to thrombosis, hypercoagulability, and microvascular damage, commonly observed in severe COVID-19. We also measured autoantibodies against intracellular proteins such as CPS1, ST3GAL6, GSTO1, ABCB9, RAB18, and NUP210 or epithelium lining components (KRT6A, KRT78, KRT84, KRT86), suggesting that these autoreactivities may derive from excessive tissue damage. Importantly, several of these proteins (GSTO1, IFIT1, SAA2) were measured in both the Chicago BALF proteomics and Munich DAC cohorts, supporting the robustness and cross-cohort generalizability of our findings on COVID-19–associated autoreactivities.

Of note, we found that the levels of several autoantibodies correlated with clinical markers of organ damage, such as elevated CRP, serum creatinine, liver transaminases, and respiratory dysfunction, pointing to a possible role as either drivers or a consequence of multiorgan dysfunction. This is in agreement with the fact that some of the autoantibodies detected in our study also appear in non–SARS-CoV-2 pneumonias or mild/severe COVID-19, suggesting that they reflect common systemic inflammation that becomes pathological especially in severe disease (63). Hence, our findings expand our understanding of COVID-19–associated autoreactivities and highlight how a diverse array of emerging cross-reactive antibodies may shape the clinical course, severity, and multiorgan failure — hallmarks of severe disease.

Our study has certain limitations. First, differences in sample procurement between cohorts may have introduced bias. The Chicago cohort was enriched for patients with severe COVID-19, as enrollment in the cohort required BALF sampling and included paired plasma samples, whereas the Munich cohort primarily analyzed serum. Associations of specific autoantibodies with clinical parameters also differed between cohorts, likely due to heterogeneous clinical management and incomplete metadata overlap. Additionally, both cohorts were relatively small, which may limit the generalizability of our findings, particularly given the high heterogeneity of autoantibody targets and the low detection frequency of specific autoantibodies in patients with severe COVID-19 (39, 64). Second, the sensitivity of the DAC assay is influenced by several factors, including low antibody titers or affinity in patient plasma, as well as the abundance and accessibility of autoantigens within the lung tissue lysate, potentially leading to missed autoantibody specificities. Moreover, our focus on lung-derived antigens may have overlooked autoantibodies targeting other organs. Finally, we were unable to assess preinfection autoantibody levels in our cohorts, limiting our ability to distinguish between preexisting autoantibodies and those induced by SARS-CoV-2 infection.

In conclusion, our data have direct implications for enhancing the clinical management of severe COVID-19. While earlier studies have proposed an autoimmune component, our work strengthens this notion by linking distinct autoantibody signatures to molecularly phenotyped plasma cell populations and defined clinical features. These autoantibodies may not only serve as biomarkers for disease monitoring, severity stratification, and rapid differential diagnosis in patients with community-acquired pneumonias but also represent potential targets to inform outcome-oriented treatment decisions in patients with PASC, such as therapeutic monitoring and pathway-targeted interventions. Future studies should explore how modulating the autoreactive immune landscape might mitigate long-term complications and refine personalized treatment strategies in COVID-19 and other postinfectious syndromes.

## Methods

### Sex as a biological variable.

The study included individuals of both sexes. No sex-specific comparisons were performed. The findings are reported for the combined cohort and are, therefore, expected to be broadly applicable to both sexes.

### Chicago cohort.

Samples from COVID-19, bacterial pneumonia, influenza, and nonpneumonia control patients were collected from participants enrolled in the SCRIPT study (STU00204868) (6). Participants were adults with clinical suspicion of pneumonia, indicated by fever, radiographic infiltrates, and respiratory secretions. All patients experienced respiratory failure requiring mechanical ventilation in the ICU. Intubation decisions were based on bedside clinicians’ assessments of worsening hypoxemia, hypercapnia, or inadequate response to high-flow oxygen or noninvasive ventilation. Extubation decisions followed a protocol-guided evaluation of spontaneous breathing and cardiorespiratory improvement. Critical care physicians at Northwestern Memorial Hospital retrospectively classified patients into 5 groups: COVID-19 pneumonia, non–COVID-19 viral pneumonia, pneumonia from other pathogens, nonpneumonia controls, and a control group, according to a standardized adjudication process. Mortality was defined as death in the hospital, lung transplantation during the hospitalization, or discharge to hospice.

BALF was screened for methicillin-resistant *Staphylococcus aureus* (MRSA) using PCR (MRSA/SA SSTI), the BioFire FilmArray Respiratory 2 (RP2) panel, and pneumonia panels. Clinical laboratory data were obtained from the Northwestern Medicine Enterprise Data Warehouse, a clinical data warehouse at Northwestern Medicine, Chicago, Illinois, USA. To focus on pneumonia type specific signatures, samples from patients with viral pneumonia (COVID-19, influenza) with bacterial superinfections at later time points were excluded from the initial analysis. This filtering resulted in 10 patients with COVID-19, 7 patients with influenza, 6 patients with bacterial pneumonia, and 7 nonpneumonia control patients (Figure 1E and Supplemental Table 1). The clinical subgroups were matched for age, race, and sex, exhibiting diverse clinical outcomes.

### Sample collection.

Bronchoscopy, often accompanied by blood draws, was performed as part of routine clinical care to guide antimicrobial therapy. BALF and plasma samples from participants in the SCRIPT study were collected between June 15, 2018, and July 6, 2020, in the ICU at Northwestern Memorial Hospital, Chicago, Illinois, USA. Clinical laboratory testing followed standard ICU protocols and included multiplex PCR (BioFire Film Array Respiratory 2 panel), automated cell counts, and urinary antigen testing for *Streptococcus pneumoniae* and *Legionella pneumophila* serogroup 1, all performed on the day of admission. For patients intubated in the ICU, BALF was collected using single-use aScope devices (Ambu, USA) under sedation and topical anesthesia. The bronchoscope was wedged into the lung area of interest based on chest imaging or procedural observations. Sequential aliquots of 30 mL normal saline were instilled and aspirated, totaling 90–120 mL. The fluid recovered from the first aliquot was discarded. BAL samples were split between clinical diagnostics and research. Nonbronchoscopic BALF (NBBALF) followed a similar procedure but was performed by a respiratory therapist using directional guidance rather than by a pulmonologist.

For patients with COVID-19, sampling targeted the lung region with the greatest radiographic abnormality and was performed by a critical care physician using a disposable device. Sedation and neuromuscular blockade were administered to prevent coughing during bronchoscopy. The earliest BALF procedures were conducted immediately after intubation to utilize the existing neuromuscular blockade.

Whole blood was collected from SCRIPT cohort patients in lithium heparin tubes on the same day as BALF or NBBALF. Plasma was separated by centrifugation at 1,690*g* for 10 minutes at 4°C and stored at −80°C until proteomic analysis. BALF and plasma samples from healthy volunteers enrolled in studies Pro00088966 and Pro00100375 at Duke University, Durham, North Carolina, USA were also collected. BALF from these volunteers was performed under sedation and topical anesthesia, guided by chest CT scans to select the bronchopulmonary segment of interest. Similar volumes of 90–120 mL saline were instilled and aspirated, discarding the first 5 mL of returned fluid.

### Munich cohort.

Serum samples of patients positive for SARS-CoV-2 collected longitudinally between March and June 2020 at the Ludwig Maximilian University of Munich Hospital (LMU), Munich, Germany (65). Sampling extended up to 54 days after admission and included patients treated in both regular wards and ICU. All serum samples were stored at −80°C in the LMU LabMed Biobank.

### Cohort stratification.

To assess patient heterogeneity and define stratification criteria within the Munich COVID-19 cohort, t-SNE was performed using the Rtsne package (v.0.16). The analysis was based on clinical metadata and blood chemistry parameters from 29 patients upon hospital admission. Only variables that did not require imputation at admission were included in the input matrix. To establish a longitudinal framework, clinical markers were profiled over time, including anti–SARS-CoV-2 N and S antibodies, CRP, creatinine (Jaffe method), lymphocyte counts, and leukocyte counts. Based on these profiles, 3 main time points were selected: day 11, day 25, and day 36. Serum aliquots from individual patients were pooled across 4 time windows: 0–11 days, 12–25 days, 26–36 days, and 36–54 days after admission. The final COVID-19 cohort included 23 patients, of whom 16 were classified as severe (ICU-admitted) and 7 as mild, based on the t-SNE clustering (Supplemental Table 8). As controls, serum samples were collected from patients admitted to the University Hospital of LMU Munich with suspected SARS-CoV-2 infection but who tested negative by PCR. These control patients were stratified based on systemic inflammation markers — CRP, creatinine, and leukocyte counts. Those within the third and fourth quartiles of the distribution for at least 1 parameter were categorized as the high-inflammatory control group, whereas those within the first and second quartiles were classified as the low-inflammatory control group. All analyses were performed using the pheatmap (v.1.0.12) and base R packages.

### Patient material processing.

Lung tissue was selected as the source for antigen profiling. A pooled lysate was generated from peritumoral lung tissue collected from 32 donors undergoing lung resection surgery for carcinoma (Supplemental Table 11). All tissue samples were obtained after transplantation and preserved under standardized conditions by the Comprehensive Pneumonology Center (CPC) BioArchive. Immediately after surgical removal, lung tissue was cut into 0.5 cm³ fragments, snap-frozen in liquid nitrogen, and stored at −80°C until further processing. Frozen lung tissue was pulverized for protein extraction using a dry tissue pulverizer (CP02, Covaris, USA). The resulting powder was resuspended in RIPA buffer (50 mM Tris-HCl pH 7.4, 150 mM NaCl, 1% Triton X-100, 0.5% sodium deoxycholate, and 1 mM EDTA, 0.1% SDS) supplemented with protease inhibitors (Complete, Roche, Switzerland). The homogenates were incubated on ice for 30 minutes and then subjected to sonication using a Bioruptor device (Diagenode, Germany) for 10 cycles (30 s on, 30 s off). Following sonication, samples were centrifuged at 18,000*g* for 5 minutes to remove insoluble debris. Protein concentration in the cleared lysates was measured using the bicinchoninic acid assay (Pierce, Thermo Fisher Scientific, Germany).

### DAC.

As previously described (31), immunoglobulins were then captured by incubating each sample with 20 μL of Protein L agarose beads (Pierce, Thermo Fisher Scientific, Germany). For antigen binding, a pooled lysate was prepared from peritumoral lung tissue collected from 32 age-matched individuals (Supplemental Table 10). A total of 1.5 g of lung lysate was aliquoted and stored; each aliquot was thawed on ice immediately prior to use. After immunoglobulin binding, beads were incubated with the lung lysate to allow antigen–antibody complex formation. The complexes were retrieved on beads, subjected to a series of washing steps to remove nonspecific binders, and subsequently eluted for downstream mass spectrometry profiling.

### Fluid sample preparation.

Plasma specimens were prepared for mass spectrometry using an automated workflow (66). Plasma proteins were denatured in a 96-well format, alkylated, and digested with Trypsin and LysC. Peptides were purified using an automated liquid handling platform (Agilent Bravo, USA). To construct a spectral library, 20 representative serum samples were pooled and fractionated into 24 fractions using high-pH reversed-phase liquid chromatography. This spectral library served as a reference for peptide identification in the experimental samples. BALF specimens were denatured in 4% SDS in Tris-HCl solution at 99°C and further processed using the Protein Aggregation Capture protocol (67). Following digestion, peptides were purified using stage-tipping with triple-layered Octadecyl–bonded (C18-bonded) silica disks. Final peptide eluates were stored at –20°C until subjected to mass spectrometry.

### Mass spectrometry.

Peptides were separated using nanoflow reversed-phase chromatography on an Evosep One liquid chromatography system (Evosep). Separation was performed on an 8 cm × 150 μm column packed with 1.9 μm ReproSil-Pur C18-AQ particles (Dr. Maisch HPLC). Two acquisition methods were applied depending on the experimental design. For general proteomics profiling, peptides were analyzed on a timsTOF Pro mass spectrometer (Bruker Daltonics) operated in data-dependent acquisition parallel accumulation–serial fragmentation (DDA-PASEF) mode over a 21-minute gradient (Evosep 60 samples-per-day [SPD] method). Each acquisition cycle included 3 PASEF scans, with accumulation and ramp times set to 100 ms each. Singly charged precursors were excluded from acquisition, the target intensity was set to 15,000, and dynamic exclusion was applied for 0.4 minutes. Quadrupole isolation widths were set to 2 Th for m/z < 700 and 3 Th for m/z > 800. The DAC samples were analyzed using the same LC system connected to a timsTOF Pro operated in data-independent acquisition parallel accumulation–serial fragmentation (DIA-PASEF) mode over a 44-minute gradient (Evosep 30 samples-per-day [SPD] method). The full scan range was set from 100 to 1,700 m/z, with a ramp time of 100 ms. Ion mobility was set between 0.7 and 1.45 1/K_0_. Each acquisition cycle consisted of 27 data-independent acquisition Parallel Accumulation–Serial Fragmentation (diaPASEF) windows followed by 1 full scan, acquired across 9 frames. The DIA windows ranged from 300 to 1,110 m/z, with an isolation window of 30 Da. Collision energy was set dynamically.

### Proteomics data processing.

Mass spectrometry raw files from data-dependent acquisition were analyzed using MaxQuant (v1.6.17.0) with the UniProt human reference proteome (UP000005640, version 2022_01). Searches were performed against the human and contaminant databases using the Andromeda search engine, applying carbamidomethylation of cysteine as a fixed modification and methionine oxidation and N-terminal acetylation as variable modifications. Peptide and protein identification was controlled at a 1% FDR, estimated using a reverse decoy database. Trypsin and LysC were defined as cleavage enzymes, allowing up to 2 missed cleavages. A minimum peptide length of 7 amino acids and at least 1 peptide per protein group were required for quantification. Precursor mass tolerance was set to 20 ppm. DAC-based raw files acquired in data-independent acquisition mode were processed with DIA-NN (v1.8.1) in library-free mode with match-between-runs enabled. Trypsin/P and LysC were used as digestion enzymes, and methionine oxidation and N-terminal acetylation were set as variable modifications. Filtering was applied at 1% FDR for precursors and gene groups, and 0.5% for the spectral library. Protein group outputs from MaxQuant and DIA-NN were filtered to retain proteins detected in at least 3 samples per group, normalized, and imputed using a normal distribution within the DEP package (v1.24.0).

### Coexpression network analysis.

Gene coexpression network analysis was applied to identify protein modules with coordinated expression patterns within each pneumonia group and characterize their functions. The analysis was conducted in R (v4.3.0) using the CoCena2 package (https://github.com/MarieOestreich/hCoCena), with log_2_-transformed, normalized, and imputed protein intensity matrices from plasma and BALF samples as input (68). Correlation cut-offs were optimized using a weighted sum approach based on Multicriteria Decision Aiding, balancing maximization of the correlation coefficient (*R²*) and number of edges/nodes while minimizing the number of independent networks (69). The resulting thresholds were 0.81 for BALF and 0.74 for plasma. The network results were clustered using the Louvain algorithm (70) with a minimum cluster size of 10 nodes. The results were presented as a mean fold change for each module and condition in a heatmap with the module/condition setting. The healthy group served as the baseline reference for calculating the group-fold changes.

### Overrepresentation analysis (ORA).

ORA was employed for each CoCena^2^ module to detect associated biological pathways. The analysis was performed using the clusterProfiler package (v.4.10.0) (71), with Reactome serving as the reference database for pathway annotation (72). For each module, the top 5 significantly enriched terms were selected, requiring a minimum of 5 detected proteins per term and an adjusted *P* < 0.05. Results were visualized as grouped dot plots, highlighting functional enrichment across modules

### Gene set variation analysis (GSVA).

GSVA was performed on imputed, log_2_-transformed protein intensity matrices to evaluate longitudinal pathway behavior and molecular term dynamics (73). Gene sets utilized for analysis included enriched Reactome pathways identified via ORA of BALF samples, along with custom-defined gene lists. Enrichment scores were computed for individual samples using the gsva R package (v1.5.0), with a minimum gene set size of 5.

### Analysis of autoreactivity data.

DAC-based proteomic data were analyzed independently for each cohort. Protein intensity values were log_2_-transformed to stabilize variance. For control groups, missing values were imputed only when fewer than 3 nonmissing values per condition were available; no imputation was performed for COVID-19 samples to avoid artificially enhancing disease-specific signals. Proteins detected in ≥ 3 patients with COVID-19 and significantly enriched relative to at least 1 control group were defined as putative autoantigens (52). patients with COVID-19 were analyzed across 4 time windows, each corresponding to the next available sample following the day of intubation. In contrast, bacterial pneumonia and influenza cohorts had only 2 time points. Control groups included high-inflammatory and low-inflammatory patients from the Munich cohort, and nonpneumonia controls from the Chicago cohort.

Differential protein abundance was assessed using Welch’s *t* test, conducted separately at each time point. Proteins with fold change > 1.3 and *P* < 0.05 were considered significantly enriched. To mitigate false positives, biological relevance was evaluated based on recurrence across patients and cohorts. Significant proteins were aggregated across time points and conditions to define cohort-specific autoantigen lists. Overlaps between cohorts were visualized using Euler diagrams generated with the Eulerr R package (v7.0.0). All analyses were performed using the Perseus software platform (v1.6.14.0) (74), and visualizations were generated using the ggplot2 package (v3.4.4).

### Modeling time and severity effects on autoreactivity.

To investigate how disease severity and time influence autoreactivity, we analyzed longitudinal proteomic data from 23 patients in the Munich cohort, comprising 1,210 log_2_-transformed protein features. Patients were grouped into *mild* and *severe* categories based on t-SNE analysis of clinical and molecular profiles. A linear mixed-effects model was applied to evaluate protein expression dynamics across 4 time points. The model was defined as follows:

*Protein_ij_* = b*_0i_* + b*_1i_* x *Time window_ij_* + b*_2_* x *Severity group_ij_* + e*_ij_*

where *Protein_ij_* is the log_2_-transformed fold change between COVID-19 and the average low inflammatory control group of expression value for sample *i* in patient *j* for a single protein. Β_oi_ is a random intercept for the *i*th patient, β_1i_ is a random slope for the *i*th patient and β_2_ is a fixed coefficient for the severity group. *Time window_ij_* and *Severity group_ij_* represent the fixed effect for the time in the *j*th observation.

Model fitting and evaluation were performed using the lme4 (v1.1-35.1), lmerTest (v3.1-3), and psych (v2.3.9) packages. Model performance was assessed using the performance package (v0.10.8), focusing on Akaike Information Criterion, Bayesian Information Criterion, Bayes Factor, and residual normality. Significance was determined using 2-way ANOVA F-tests on fixed effects, with FDR-adjusted *P* < 0.05 considered significant.

### Association analysis.

To investigate potential associations between autoantigen presence and clinical outcomes in patients with severe COVID-19, we performed Wilcoxon signed-rank tests. Patients positive for each autoantigen were compared with those negative for the same antigen. Results were visualized as a heatmap, with significant associations (*P* < 0.05) highlighted in red. Tests were omitted and marked in dark grey when either group had fewer than 3 observations (*n* < 3) or when autoantigen-positive cases were insufficient for statistical analysis. All analyses were performed in R (v4.3.2) using the ggpubr (v0.6.0), stats (v3.6.2), ggplot2, and pheatmap packages.

### Single-cell RNA-seq data analysis.

Single-cell multi-omics data of peripheral blood immune cells from hospitalized patients with COVID-19 and healthy controls were retrieved from ArrayExpress (E-MTAB-10026) in the.h5ad format. The dataset contained transcriptomic raw counts, surface protein data, and immune receptor sequences (24). LPS-stimulated and hospitalized patients without COVID-19 were excluded from the original object using the Scanpy (v.1.9.1) package, resulting in 102 patients with COVID-19.

Subsequent preprocessing and analysis were performed in Seurat (v4.2.1). Cells with > 10% mitochondrial content or < 1,000 detected genes were filtered using *subset()*. Data were normalized with *NormalizeData()* using the “LogNormalize” method (scale factor = 10,000) and scaled via *ScaleData()*. Highly variable genes (HVGs) were identified using *scib.preprocessing.hvg_batch()* from scvi-tools (v0.6.8), with patient ID as the batch factor and a 15% target gene fraction. Genes with zero expression were excluded. Harmony (v0.1.1) was used to correct principal components (PCs) for patient-specific batch effects. UMAP embeddings and the neighborhood graph were computed, and clustering was performed using the Louvain algorithm with a resolution of 0.5 and 30 PCs.

Differentially expressed genes (DEGs) for initial clustering were computed using the MAST algorithm (75). Clusters were annotated using canonical PBMC markers (76). B and plasma cells were identified based on expression of marker genes (*CD79A*, *MS4A1*, *CD19*, *CD22*, *JCHAIN*, *IGKC*) and multiple IGH/IGA subclasses (*IGHG1*, *IGHG2*, *IGHG3*, *IGHG4*, *IGHA1*), and they were subsetted for further analysis. HVGs were recalculated for the subset, followed by data scaling, PCA, and batch correction using Harmony.

### B and plasma cell annotation.

UMAP embeddings were recalculated using 20 PCs, a resolution of 0.5, and Harmony for batch correction to refine B and plasma cell subset separation. DEGs were identified per cluster using Seurat’s *FindAllMarkers()* function with the MAST algorithm (75). Significant DEGs were defined by a log fold change > 0.25, expression in ≥ 25% of cells, and Bonferroni-adjusted *P* < 0.05. The top 20 DEGs per cluster were used for manual annotation based on known B and plasma cell markers from the literature (77). The CITE-seq data were normalized using centered log ratio normalization via the *NormalizeData()* function (78). Surface protein markers were identified using *FindAllMarkers()* (MAST algorithm) applied to the protein assay. Cluster annotations were refined using known markers from Woodruff et al. (25) and B1 cell–specific markers (79).

### Single-cell BCR-seq analysis.

The single-cell V(D)J dataset from (24) were retrieved from ArrayExpress (E-MTAB-10026) and were processed following the *Single-cell Best Practices* guidelines (80). Filtered contig annotations were analyzed using Scirpy (v0.11.2) in Python (v3.10), retaining only cells with a single, complete B cell receptor (BCR), and excluding those with incomplete or multiple chains. Filtered contigs were merged with the single-cell B and plasma cell object using *ir.pp.merge_with_ir()*, excluding IR-negative cells, resulting in 38,006 BCR^+^ cells. Clonotypes were assigned using Dandelion (v0.3.0), which accounts for somatic hypermutation by comparing all possible synonymous 5-mer variants. A distance threshold for clonotype assignment was determined using *ddl.pp.calculate_threshold()* based on the bimodal distribution of sequence similarities. Clonotyping was performed across the entire dataset. Downstream analyses, including clonotype frequency by disease severity and gene segment usage, were conducted using Scirpy and Seurat (v4.2.1).

### Statistics.

Clinical metadata and clinical blood chemistry parameters were analyzed and visualized in R (v4.3.0). Group comparisons were performed using base R and the tidyverse package (v1.3.0), with visualization via ggplot2 (v3.4.4) and ggpubr (v0.6.0). Normality of continuous variables was assessed using the Shapiro-Wilk test. For nonnormally distributed data, nonparametric tests were applied, including the Wilcoxon rank-sum test for 2-group comparisons and the Kruskal-Wallis test with Dunn’s post hoc corrections for multigroup comparisons. All statistical tests were 2-sided, and *P* < 0.05 were considered statistically significant.

### Study approval.

The Chicago cohort received ethical approval from the Northwestern University IRB, and informed consent was obtained from all participants or their legal representatives. The protocol and anonymized data analysis of the Munich cohort were approved by the Ethics Committee of LMU Munich (reference no. 21-0047). All procedures complied with the ethical principles of the World Medical Association Declaration of Helsinki and the U.S. Department of Health and Human Services Belmont Report.

### Data availability.

All of the data from this study are presented in the article supplemental material, Supporting Data Values file, and public repositories. Raw proteomic data and MaxQuant output tables are available from the PRIDE repository (81) under the accession nos. PXD051341 (Chicago cohort: BALF, and plasma) and PXD051982, PXD051948 (Chicago and Munich cohorts: DAC). The analysis code supporting this study is publicly available at https://github.com/schillerlab/2025_AnnaSemenova_Autoantibody; commit ID code_paper_COVID_231125.

## Author contributions

GRSB, TSK, and HBS designed the study; TAP curated clinical sample collection; AS, JBMR, PG, and MMH conducted experiments; AS and SRSP acquired and analyzed data; RAG, LR, LMH, DT, MM, AÖY, RGW, AVM, BDS, GRSB, and HBS provided resources; AS, TSK, and HBS wrote the manuscript. All authors read and approved the manuscript.

## Conflict of interest

The authors have declared that no conflict of interest exists.

## Funding support

This work was supported in part by the NIH. In accordance with the NIH Public Access Policy, the manuscript will be made publicly available in PubMed Central.

- NIH (grant nos. U19 AI135964, R01 HL149883-01, U01 TR003528, R01 AI158530, P01 HL154998) and FDA (75F40122C00134) (RGW)
- NIH (grant nos. U19 AI135964, P01 AG049665, P01 HL154998, P01 HL169188, U19 AI181102, R01 HL153312, R01 HL158139, and R01 ES034350) and research grants from AbbVie and Merck (AVM)
- NIH awards R01 HL149883, R01 HL153122, P01 HL154998, P01 AG049665, U19 AI135964, and U19 AI181102 (BDS)
- Simpson Querrey Lung Institute for Translational Science, the NIH (grant nos. P01 AG049665, P01 HL154998, U54 AG079754, R01 HL147575, R01 HL158139, R01 HL147290, R21 AG075423 and U19 AI135964), and the Veterans Administration (award no. I01CX001777) (GRSB)
- Helmholtz Association (Network grant CoViPa - Virological and immunological determinants of COVID-19 pathogenesis - lessons to get prepared for future pandemics), as well as the German Center for Lung Research (DZL) (HBS)

## Supplementary Material

Supplemental data

Supplemental table 11

Supplemental table 2

Supplemental table 3

Supplemental table 4

Supplemental table 5

Supplemental table 6

Supplemental table 7

Supplemental table 8

Supplemental tables and 10

Supporting data values

## Version 1 06/08/2026

Electronic publication
