# Supplementary material for: Longitudinal clinical proteomics reveals pneumonia type–specific protein biomarkers and autoantibodies
Source: JCI Insight. 2026 Jun 8;11(11):e203056. doi: 10.1172/jci.insight.203056 (PMC13313542; doi:10.1172/jci.insight.203056)
Supplement: Supplemental data [file jciinsight-11-203056-s251.pdf]

Supplemental figure legends

Figure S1 - related to Figure 1. (a-b) Network plot of representative (a) bacterial-specific and (b) COVID-19-specific BALF protein modules. The circle size of the terms corresponds to the number of included proteins. (c) Pearson correlation of neutrophil degranulation proteins and normalized neutrophil frequency in the BALF of bacterial pneumonia patients. (d-e) Box plots displaying the longitudinal enrichment of (d) platelet activation signaling and aggregation and (e) regulation of complement cascade Reactome term proteins in BALF specimens across the four pneumonia types. Each dot represents the enrichment score for an individual patient. Data are represented as mean ± SD and were statistically assessed with the non-parametric Kruskal-Wallis test. Proteins involved in the term are displayed on the right side of the plot.

Figure S2 - related to Figure 2. (a) Network plots of representative bacterial-specific, COVID-19-specific and influenza-specific plasma protein modules. The circle size of the terms corresponds to the number of included proteins. (b) Scatter plot displaying immunoglobulin segment abundance in the BALF (x-axis) and plasma (y-axis) of Influenza patients at the time point upon intubation. Dot size corresponds to the number of patients that express a detected protein.

Figure S3 - related to Figure 3. (a) UMAP plot of 632.209 PBMCs from 90 COVID-19 patients and 23 healthy controls, colored by annotation [24]. B and plasma cell clusters are circled. (b) Dot plot of the top 10 unique gene markers for each identified B cell cluster. (c) Dot plot of differentially expressed surface protein markers for each identified B cell cluster. (d) Dot plot of B1 B cell-specific surface protein markers for each identified B cell cluster. (e) Bar plots showing the isotype distribution of B and plasma cell populations across COVID-19 disease severity groups. (f) Box plots showing the proportion of B and plasma cell populations across COVID-19 disease severity groups. Each dot represents an individual patient sample. Data are represented as mean ± SD and were statistically assessed with the non-parametric Kruskal-Wallis test. (g) UMAP visualization of 38,063 peripheral

blood B/plasma cells from 90 COVID-19 patients and 23 healthy controls, colored by the expression of proteomics-identified V-segment clonotypes.

Figure S4 - related to Figure 4. (a) Schema exhibiting the criteria of selection of control low inflammatory (n=24) and control high inflammatory (n=23) groups from SARS-CoV-2 negative patients (n=264) enrolled in the University Hospital of LMU. (b-c) Box plots displaying the longitudinal enrichment of (b) BALF and (e) shared BALF/plasma segments in BALF specimens across the four pneumonia types. Each dot represents the enrichment score for an individual patient. Data are represented as mean ± SD and were statistically assessed with the non-parametric Kruskal-Wallis test. (d-e) Volcano plots displaying immunoglobulin segment abundance between pneumonia types and controls in (d) the Chicago and (e) the Munich cohort at the time point upon intubation. Dot size corresponds to the number of patients that express a detected protein.

Figure S5 - related to Figure 5. (a) Secretion levels of circulating IL-6 and C-reactive protein in the two COVID-19 patient clusters of the Munich cohort. (b) Longitudinal assessment of clinical parameters for the two COVID-19 patient clusters of the Munich cohort. Three time windows were subsequently defined: 0-11 days, 12-36 days, 37-56 days.

Figure S6 - related to Figure 6. (a-b) Heatmaps presenting associations between the detection of cohort-specific putative autoantibodies in the peripheral blood of (a) COVID-19 patients (n=13) of the Chicago and (b) severe COVID-19 patients (n=16) of the Munich cohort upon intubation on the x-axis and selected clinical parameters on the y-axis. The color denotes the output of the Wilcoxon test (p-value). The dark grey color corresponds to the absence of the test results due to missing values. (c-d) Box plots showing the significant associations between putative shared autoantigens and clinical parameters in two cohorts. The x-axis represents COVID-19 patients categorized based on the presence (+) or absence (-) of detected autoantigens at the time of intubation. Statistical significance was assessed using the Wilcoxon test.

Supplemental tables

Table S1. Baseline characteristics of Chicago on the initial day of the intubation

Table S2. BALF protein modules hCocena

Table S3. ORA analysis of the Reactome terms in BALF

Table S4. Plasma protein modules hCocena

Table S5. ORA analysis of the Reactome terms in plasma

Table S6. Transcriptomic markers of B and plasma cell subsets

Table S7. Transcriptomic markers of dividing plasmablast and IgA high plasma cell clusters

Table S8. Baseline characteristics of the Munich cohort at the first time window (0-11 days)

Table S9. Master list of autoantigens from the Chicago and Munich cohort

Table S10. Patient metadata for peritumoral lung tissues

Figure S1

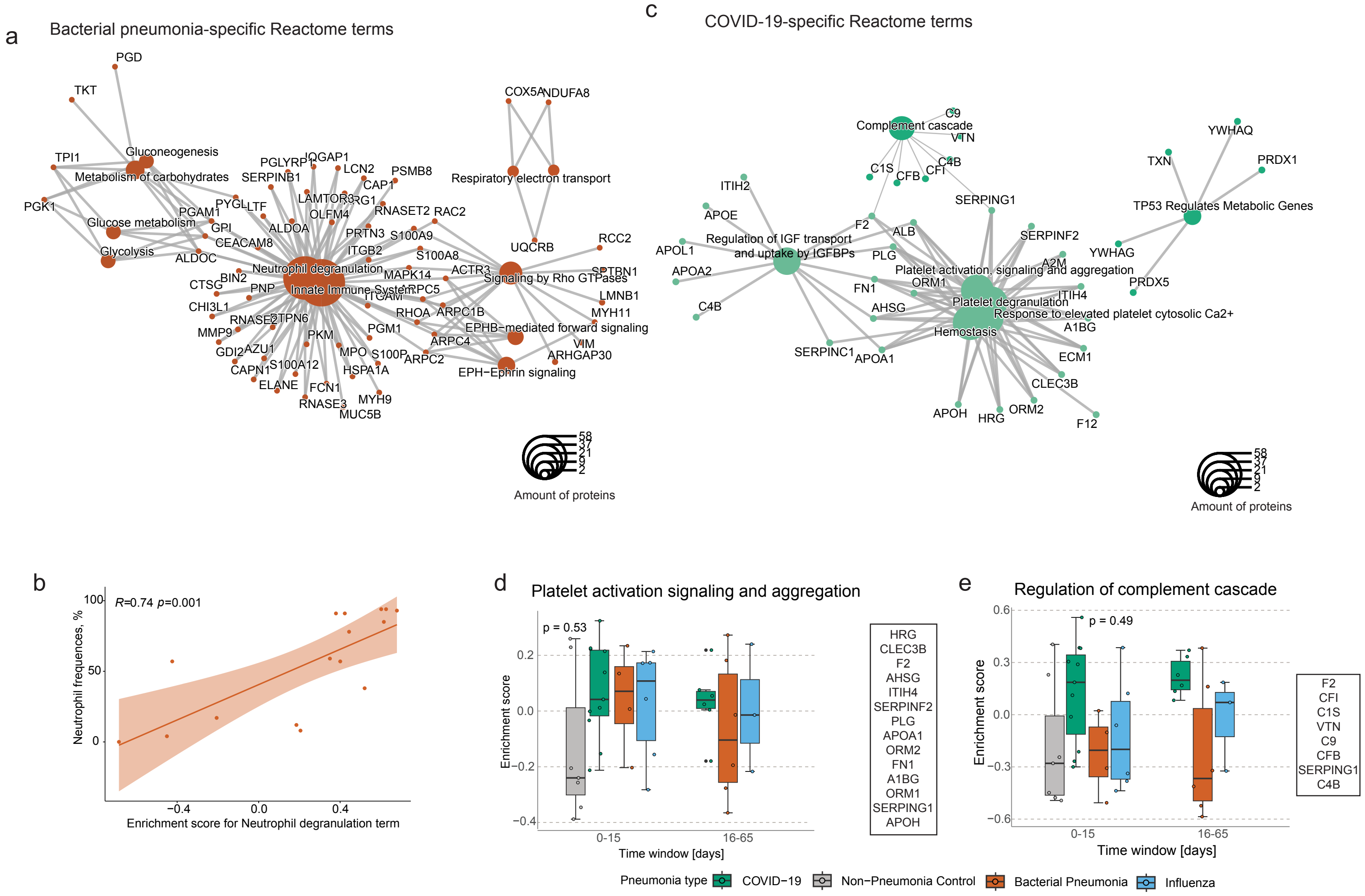
a
Bacterial pneumonia-specific Reactome terms
Gluconeogenesis
Metabolism of carbohydrates
Glucose metabolism
Glycolysis
Neutrophil degranulation
Innate Immune System
Respiratory electron transport
Signaling by Rho GTPases
EPHB−mediated forward signaling
EPH−Ephrin signaling
Amount of proteins
c
COVID-19-specific Reactome terms
Complement cascade
Regulation of IGF transport and uptake by IGFBPs
TP53 Regulates Metabolic Genes
Platelet activation, signaling and aggregation
Platelet degranulation
Response to elevated platelet cytosolic Ca2+
Hemostasis
Amount of proteins
b
R=0.74 p=0.001
Neutrophil frequences, %
Enrichment score for Neutrophil degranulation term
d
Platelet activation signaling and aggregation
p = 0.53
Enrichment score
Time window [days]
0-15
16-65
HRG
CLEC3B
F2
AHSG
ITIH4
SERPINF2
PLG
APOA1
ORM2
FN1
A1BG
ORM1
SERPING1
APOH
e
Regulation of complement cascade
p = 0.49
Enrichment score
Time window [days]
0-15
16-65
F2
CFI
C1S
VTN
C9
CFB
SERPING1
C4B
Pneumonia type
COVID−19
Non−Pneumonia Control
Bacterial Pneumonia
Influenza

Figure S2

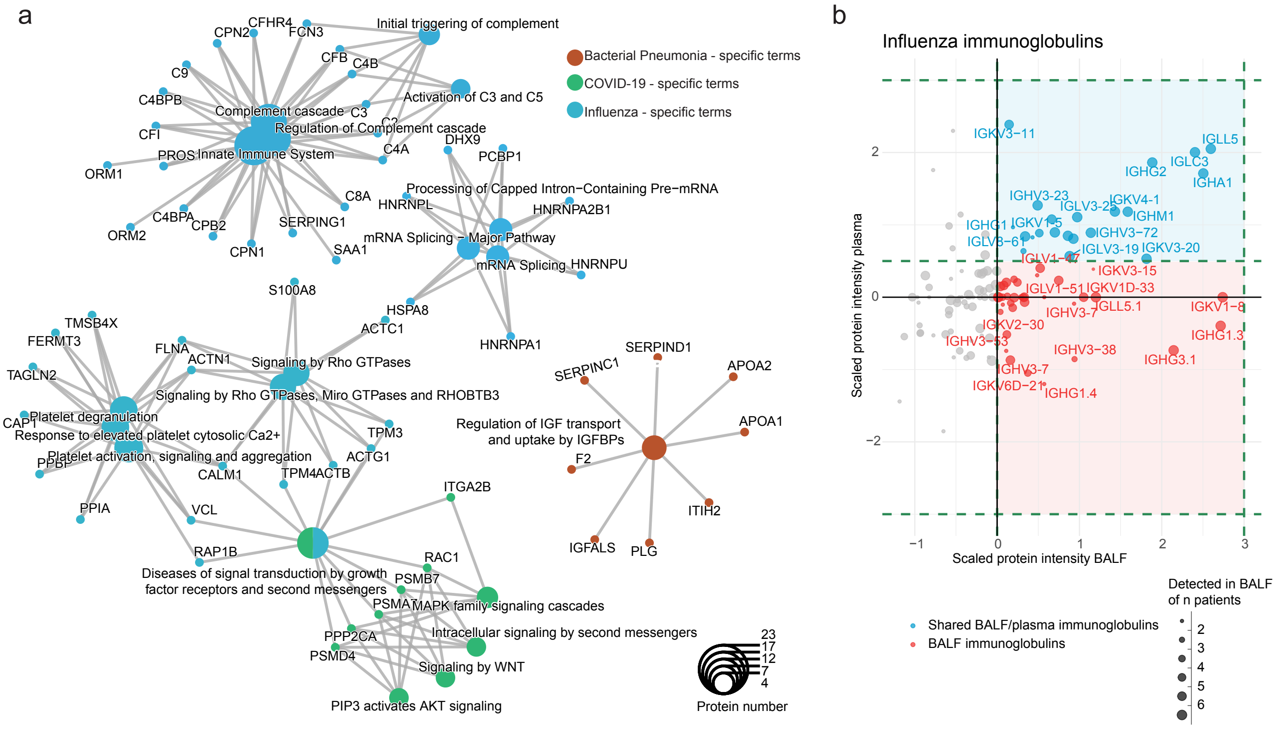

a
Influenza-specific Reactome terms
Bacterial Pneumonia - specific terms
COVID-19 - specific terms
Influenza - specific terms
Initial triggering of complement
Complement cascade
Regulation of Complement cascade
Activation of C3 and C5
Innate Immune System
Processing of Capped Intron−Containing Pre−mRNA
mRNA Splicing − Major Pathway
mRNA Splicing
Signaling by Rho GTPases
Signaling by Rho GTPases, Miro GTPases and RHOBTB3
Platelet degranulation
Response to elevated platelet cytosolic Ca2+
Platelet activation, signaling and aggregation
Regulation of IGF transport and uptake by IGFBPs
Diseases of signal transduction by growth factor receptors and second messengers
MAPK family signaling cascades
Intracellular signaling by second messengers
Signaling by WNT
PIP3 activates AKT signaling
Protein number
23
17
12
7
4
b
Influenza immunoglobulins
Scaled protein intensity BALF
Scaled protein intensity plasma
Shared BALF/plasma immunoglobulins
BALF immunoglobulins
Detected in BALF of n patients

Figure S3

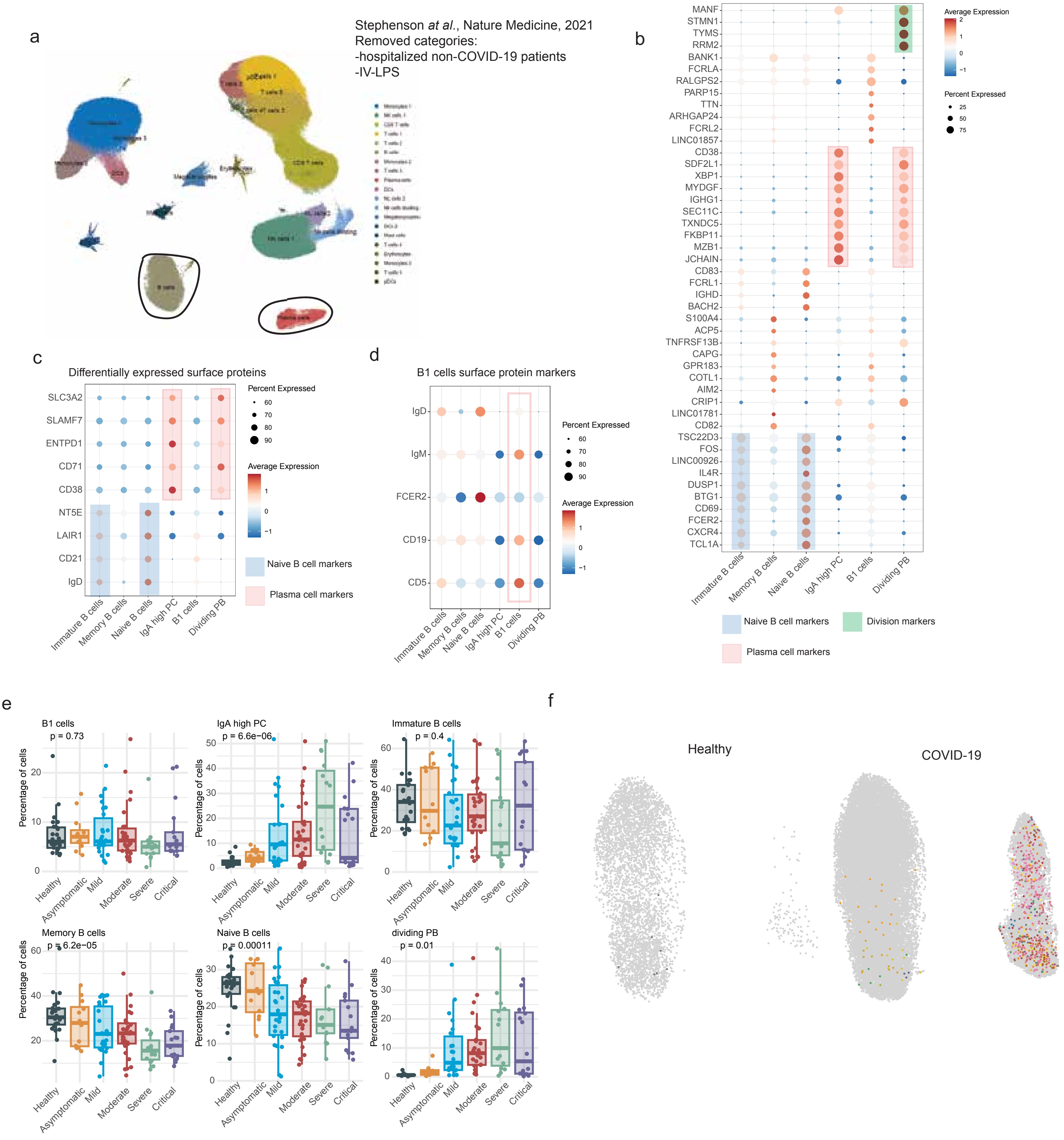
a
Stephenson at al., Nature Medicine, 2021
Removed categories:
-hospitalized non-COVID-19 patients
-IV-LPS
b
Differentially expressed genes
MANF
STMN1
TYMS
RRM2
BANK1
FCRLA
RALGPS2
PARP15
TTN
ARHGAP24
FCRL2
LINC01857
CD38
SDF2L1
XBP1
MYDGF
IGHG1
SEC11C
TXNDC5
FKBP11
MZB1
JCHAIN
CD83
FCRL1
IGHD
BACH2
S100A4
ACP5
TNFRSF13B
CAPG
GPR183
COTL1
AIM2
CRIP1
LINC01781
CD82
TSC22D3
FOS
LINC00926
IL4R
DUSP1
BTG1
CD69
FCER2
CXCR4
TCL1A
Average Expression
Percent Expressed
Immature B cells
Memory B cells
Naive B cells
IgA high PC
B1 cells
Dividing PB
Naive B cell markers
Division markers
Plasma cell markers
c
Differentially expressed surface proteins
SLC3A2
SLAMF7
ENTPD1
CD71
CD38
NT5E
LAIR1
CD21
IgD
Percent Expressed
Average Expression
Naive B cell markers
Plasma cell markers
d
B1 cells surface protein markers
IgD
IgM
FCER2
CD19
CD5
Percent Expressed
Average Expression
e
B1 cells
p = 0.73
IgA high PC
p = 6.6e−06
Immature B cells
p = 0.4
Memory B cells
p = 6.2e−05
Naive B cells
p = 0.00011
dividing PB
p = 0.01
Percentage of cells
Healthy
Asymptomatic
Mild
Moderate
Severe
Critical
f
Healthy
COVID-19

Figure S4

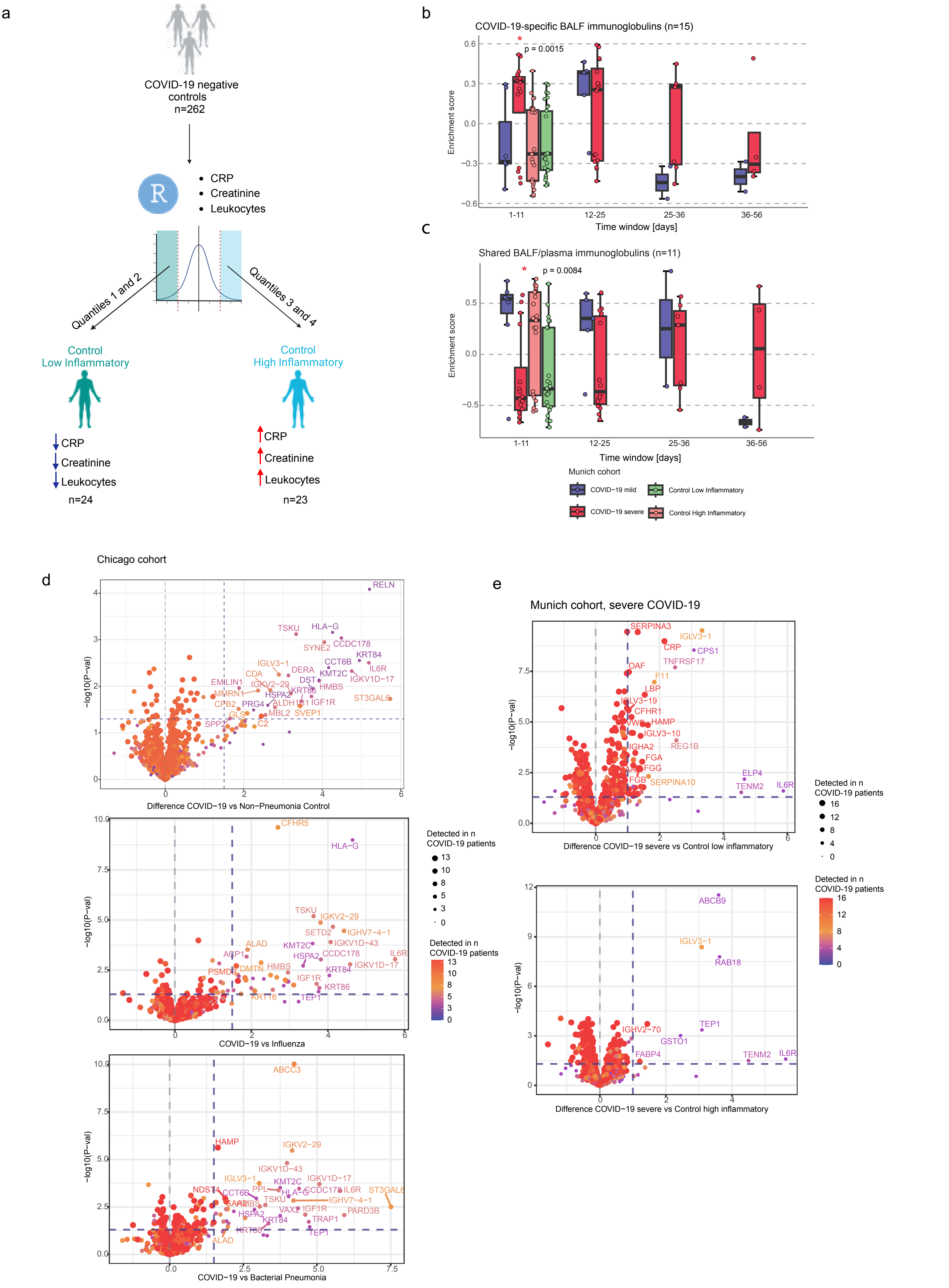
a
COVID-19 negative controls n=262
CRP
Creatinine
Leukocytes
Quantiles 1 and 2
Quantiles 3 and 4
Control Low Inflammatory
Control High Inflammatory
CRP
Creatinine
Leukocytes
n=24
CRP
Creatinine
Leukocytes
n=23
b
COVID-19-specific BALF immunoglobulins (n=15)
p = 0.0015
Enrichment score
Time window [days]
1-11
12-25
25-36
36-56
c
Shared BALF/plasma immunoglobulins (n=11)
p = 0.0084
Enrichment score
Time window [days]
Munich cohort
COVID−19 mild
Control Low Inflammatory
COVID−19 severe
Control High Inflammatory
Chicago cohort
d
−log10(P−val)
Difference COVID−19 vs Non−Pneumonia Control
COVID−19 vs Influenza
COVID−19 vs Bacterial Pneumonia
Detected in n COVID-19 patients
e
Munich cohort, severe COVID-19
Difference COVID−19 severe vs Control low inflammatory
Difference COVID−19 severe vs Control high inflammatory
Detected in n COVID-19 patients

Figure S5

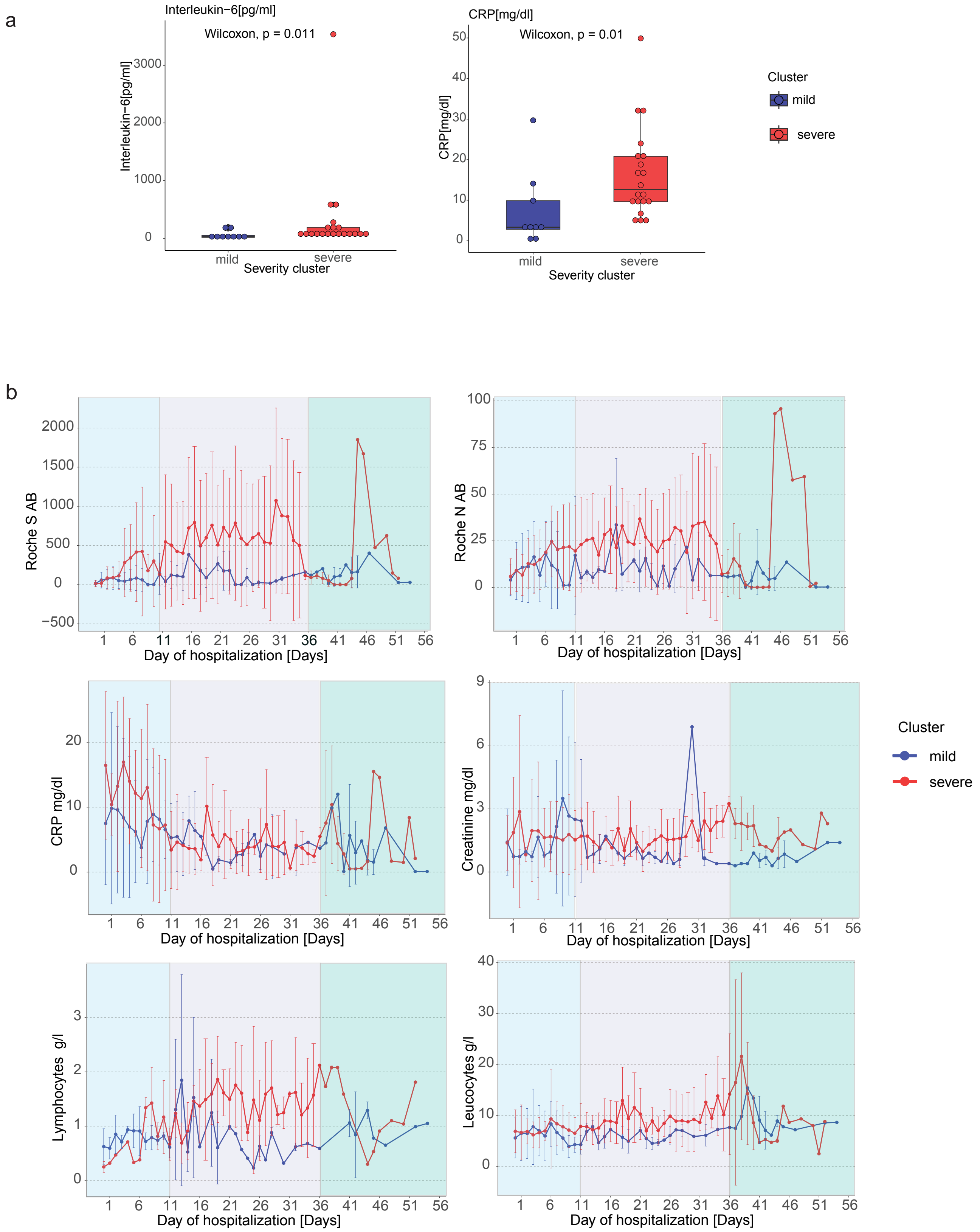
a
Interleukin−6[pg/ml]
Wilcoxon, p = 0.011
Interleukin−6[pg/ml]
Severity cluster
mild
severe
CRP[mg/dl]
Wilcoxon, p = 0.01
CRP[mg/dl]
Severity cluster
Cluster
mild
severe
b
Roche S AB
Roche N AB
CRP mg/dl
Creatinine mg/dl
Lymphocytes g/l
Leucocytes g/l
Day of hospitalization [Days]
Cluster
mild
severe

Figure S6

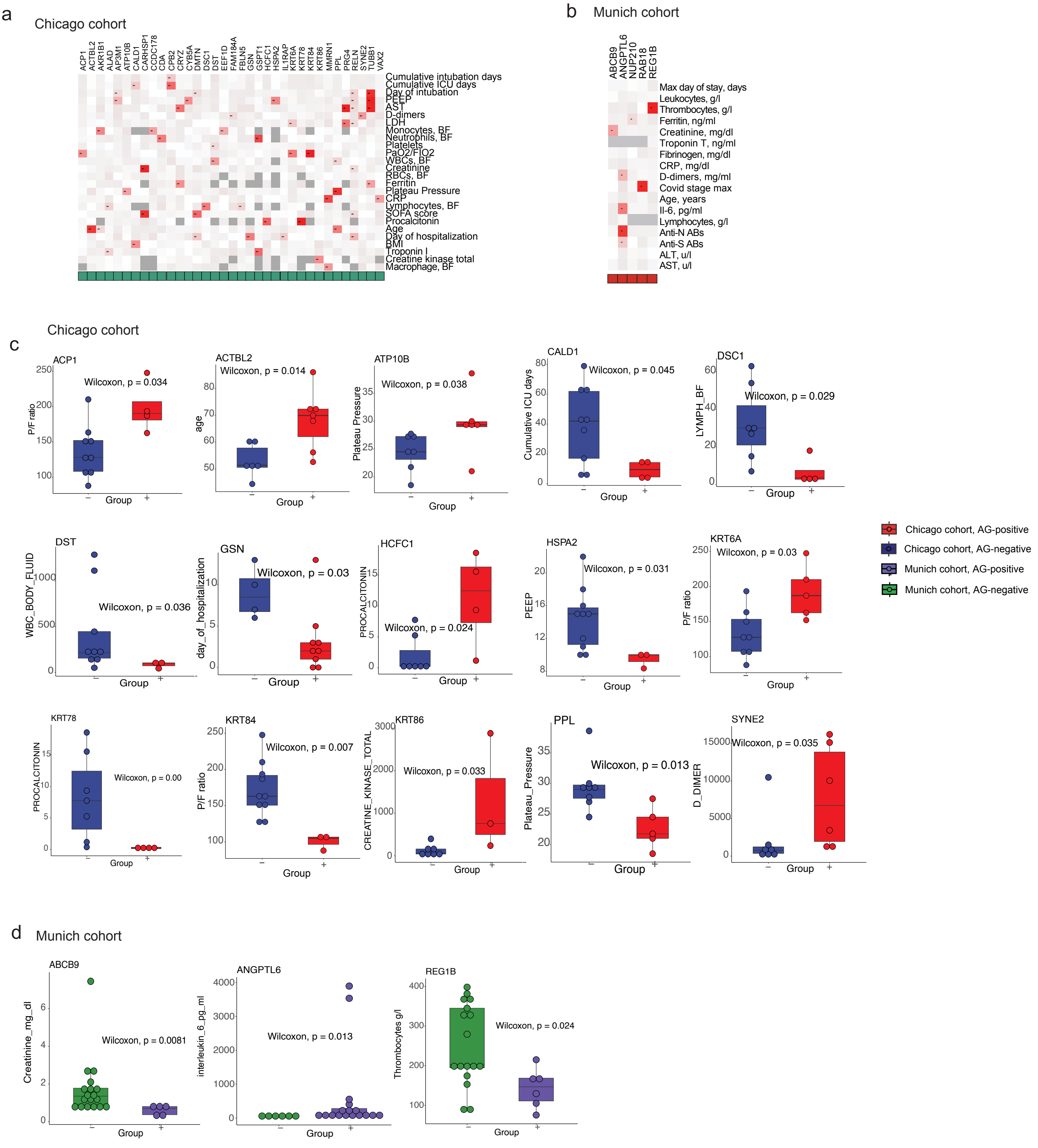

a
Chicago cohort
ACP1
ACTBL2
AKR1B1
ALAD
AP3M1
ATP10B
CALD1
CARHSP1
CCDC178
CDA
CPB2
CRYZ
CYB5A
DMTN
DSC1
DST
EEF1D
FAM184A
FBLN5
GSN
GSPT1
HCFC1
HSPA2
IL1RAP
KRT6A
KRT78
KRT84
KRT86
MMRN1
PPL
PRG4
RELN
SYNE2
TUBB1
VAX2
Cumulative intubation days
Cumulative ICU days
Day of intubation
PEEP
AST
D-dimers
LDH
Monocytes, BF
Neutrophils, BF
Platelets
PaO2/FIO2
WBCs, BF
Creatinine
RBCs, BF
Ferritin
Plateau Pressure
CRP
Lymphocytes, BF
SOFA score
Procalcitonin
Age
Day of hospitalization
BMI
Troponin I
Creatine kinase total
Macrophage, BF
b
Munich cohort
ABCB9
ANGPTL6
NUP210
RAB18
REG1B
Max day of stay, days
Leukocytes, g/l
Thrombocytes, g/l
Ferritin, ng/ml
Creatinine, mg/dl
Troponin T, ng/ml
Fibrinogen, mg/dl
CRP, mg/dl
D-dimers, mg/ml
Covid stage max
Age, years
Il-6, pg/ml
Lymphocytes, g/l
Anti-N ABs
Anti-S ABs
ALT, u/l
AST, u/l
c
Chicago cohort
ACP1
P/F ratio
Wilcoxon, p = 0.034
ACTBL2
age
Wilcoxon, p = 0.014
ATP10B
Plateau Pressure
Wilcoxon, p = 0.038
CALD1
Cumulative ICU days
Wilcoxon, p = 0.045
DSC1
LYMPH_BF
Wilcoxon, p = 0.029
DST
WBC_BODY_FLUID
Wilcoxon, p = 0.036
GSN
day_of_hospitalization
Wilcoxon, p = 0.03
HCFC1
PROCALCITONIN
Wilcoxon, p = 0.024
HSPA2
PEEP
Wilcoxon, p = 0.031
KRT6A
P/F ratio
Wilcoxon, p = 0.03
KRT78
PROCALCITONIN
Wilcoxon, p = 0.00
KRT84
P/F ratio
Wilcoxon, p = 0.007
KRT86
CREATINE_KINASE_TOTAL
Wilcoxon, p = 0.033
PPL
Plateau_Pressure
Wilcoxon, p = 0.013
SYNE2
D_DIMER
Wilcoxon, p = 0.035
Group
Chicago cohort, AG-positive
Chicago cohort, AG-negative
Munich cohort, AG-positive
Munich cohort, AG-negative
d
Munich cohort
ABCB9
Creatinine_mg_dl
Wilcoxon, p = 0.0081
ANGPTL6
interleukin_6_pg_ml
Wilcoxon, p = 0.013
REG1B
Thrombocytes g/l
Wilcoxon, p = 0.024
Group

Table S1: Baseline characteristics of cohort 1 on the initial day of the intubation

	**COVID-19 n=13**	**Non-pneumonia control n=7**	**Bacterial pneumonia n=6**	**Influenza n=7**	**P-value**
Age	61.05 ± 12.7	66.54 ± 5.3	59.59 ± 15.4	58.84 ± 11.2	0.612
Sex	10 male (77%), 3 female (23%)	3 male (43%), 4 female (57%)	4 male (67%), 2 female (33%)	4 male (57%), 3 female (43%)	-
Day of intubation [days]	3.70 ± 6.1	5.57 ± 5.0	10.00 ± 10.6	4.14 ± 7.9	0.139
ARDS	13 (100%)	7 (100%)	6 (100%)	7 (100%)	-
Deceased	2 (15%)	3 (43%)	4 (67%)	4 (57%)	-
Superinfection	3 (23%)	NA	NA	0 (0%)	-
Smoking status	4 never smoker (31%), 4 past smoker (31%), 5 NA (38%)	5 never smoker (71%), 2 past smoker (29%)	3 never smoker (50%), 1 past smoker (17%), 2 NA (33%)	5 never smoker (71%), 2 past smoker (29%)	-
Cumulative ICU days [days]	20.80 ± 19.9	11.71 ± 5.6	25.00 ± 11.9	16.86 ± 8.6	0.325
PaO2/FIO2 ratio	164.36 ± 5.6	248.29 ± 5.3	212.50 ± 60.0	208.93 ± 68.9	0.184
SOFA score	9.30 ± 4.5	9.86 ± 4.5	12.60 ± 5.4	12.57 ± 3.8	0.374
Bacterial strains	*Pseudomonas aeruginosa* (3), *Enterobacter aerogenes* (2), *Enterobacter cloacae* complex (1), *Citrobacter koseri* (1), *Achromobacter xylosoxidans* (1), *Enterococcus faecalis* (1)	NA	*Enterobacter cloacae* complex (1), *Enterobacter aerogenes* (1), *Pseudomonas aeruginosa* (2), *Enterococcus* (species unspecified) (1), *Viridans streptococci* (2)	*Klebsiella pneumoniae* (1), *Pseudomonas aeruginosa* (1), *Enterococcus* (species unspecified) (1)	
Viral strains	NA	NA	NA	Human rhinovirus / Enterovirus (1), Influenza A virus (1), Influenza A virus subtype H1N1 (1),	

				Human parainfluenza virus 4 (1)	

Table S9: Baseline characteristics of the Munich cohort at the first time window (0-11 days)

	**Mild n=7**	**Severe n=16**
Age	66.3±19	66.4±11.1
Sex	6 male (86%), 1 female (14%)	13 male (81%), 3 female (19%)
ARDS	0 (0%)	16 (100%)
ECMO	0 (0%)	3 (18.8%)
Deceased	0 (0%)	6 (37.5%)
Immunosuppression	2 (28.6%)	9 (56.2%)
Acute Kidney Injury	2 (28.6%)	12 (75%)
Length of hospitalization [days]	33.4±12.9	32.6±11.9
CRP [mg/dl]	7.0±6.8	13.3±9.0
Interleukin 6 [pg/ml]	52.9±32.6	602.2±1188.0
